# Emerging roles of metabolic biomarkers in renal cell carcinoma: from molecular mechanisms to clinical implications

**DOI:** 10.3389/fcell.2025.1664292

**Published:** 2025-09-23

**Authors:** Junkai Yang, Daojia Miao, Xinwei Li, Chuanyi Zhao, Diaoyi Tan, Songming Wu, Feiyi Lu, Jian Shi, Qingyang Lv, Hailong Ruan, Zhiyong Xiong, Xiaoping Zhang

**Affiliations:** ^1^ Department of Urology, Union Hospital, Tongji Medical College, Huazhong University of Science and Technology, Wuhan, China; ^2^ Institute of Urology, Union Hospital, Tongji Medical College, Huazhong University of Science and Technology, Wuhan, China; ^3^ Shenzhen Huazhong University of Science and Technology Research Institute, Shenzhen, China

**Keywords:** renal cell carcinoma, metabolic reprogramming, metabolic biomarkers, targeted therapy, RCC, biomarker, cancer

## Abstract

Renal cell carcinoma (RCC) is a common malignancy of the urinary system. Due to its asymptomatic nature in the early stages, many patients present with advanced or metastatic disease at the time of diagnosis. Existing therapeutic strategies for advanced RCC exhibit limited efficacy, underscoring the urgent need for novel therapeutic approaches. Recently, metabolic reprogramming—characterized by alterations in glucose metabolism, lipid synthesis, and amino acid metabolism—has emerged as a critical biological adaptation enabling tumor cell proliferation and survival within the tumor microenvironment. This review introduces the major metabolic reprogramming mechanisms in RCC, including enhanced glycolysis, augmented lipid synthesis, and altered amino acid metabolism. We summarize the associations between RCC progression and key metabolic molecules involved in these pathways, highlighting their potential clinical value as diagnostic markers, prognostic indicators, and therapeutic targets. To date, most studies have focused primarily on describing the correlations between metabolic dysregulation and tumor progression or therapeutic resistance in RCC. However, the molecules and pathways involved in these metabolic processes also represent promising targets for metabolic interventions. In this context, we further propose novel therapeutic strategies targeting key metabolic nodes such as HIF-2α, GLUT and FASN, offering new insights into precision treatment approaches for RCC.

## 1 Introduction

**FIGURE 1:**
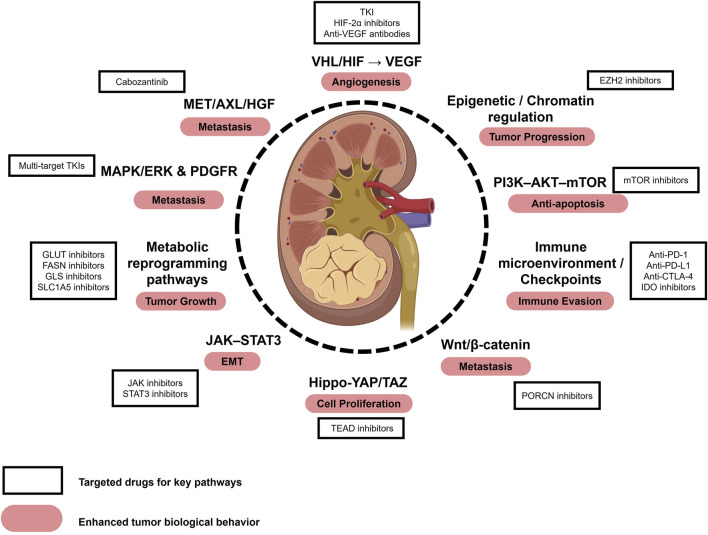
Key pathways, biological behaviors, and therapeutic targets in RCC. Major signaling pathways implicated in RCC include the VHL/HIF–VEGF axis, PI3K–AKT–mTOR, MET/AXL/HGF, MAPK/ERK and PDGFR, immune checkpoint pathways, metabolic reprogramming, JAK–STAT3, epigenetic/chromatin regulation, Hippo–YAP/TAZ, and Wnt/β-catenin. These pathways contribute to enhanced tumor biological behaviors such as angiogenesis, epithelial–mesenchymal transition (EMT), and metastasis. Representative therapeutic agents targeting these pathways include tyrosine kinase inhibitors (TKIs), HIF-2α inhibitors, mTOR inhibitors, and anti-VEGF antibodies, some of which are already used in clinical practice while others remain under investigation. Abbreviations: EMT, Epithelial-Mesenchymal Transition; TKI, Tyrosine Kinase Inhibitor.

Renal cell carcinoma (RCC) is one of the most common malignancies of the urinary system, accounting for approximately 2%–3% of all adult cancers (Catalano et al., 2023). Pathologically, RCC is classified into several subtypes, including clear cell RCC (ccRCC), papillary RCC, chromophobe RCC, translocation-associated RCC, medullary RCC, and collecting duct RCC, with ccRCC being the predominant subtype, representing 75%–85% of all RCC cases (Tykodi et al., 2022). In 2023, it was estimated that approximately 81,800 new RCC cases and 14,890 related deaths would occur in the United States (Siegel et al., 2024). Due to its asymptomatic nature in the early stages, RCC is often diagnosed at an advanced or metastatic stage, significantly compromising patient survival and prognosis. Current therapeutic approaches for RCC include surgical resection, targeted therapies, and immune checkpoint inhibitor (ICI) therapies (Srivastava et al., 2022). Targeted therapies comprise tyrosine kinase inhibitors (TKIs), hypoxia-inducible factor (HIF) pathway inhibitors, mTOR inhibitors, and anti-angiogenic agents such as bevacizumab. Immunotherapy centers on ICIs, most notably inhibitors targeting PD-1/PD-L1, and CTLA-4. However, effective treatment options remain limited for patients with advanced or drug-resistant RCC, underscoring the urgent need for novel biomarkers and therapeutic targets to enhance disease management. Several key oncogenic pathways, such as the VHL/HIF axis, PI3K–AKT–mTOR, MET/AXL, MAPK/ERK, immune checkpoints, and metabolic reprogramming, have been implicated in RCC progression and metastasis, with corresponding inhibitors already in clinical use or under investigation (Figure 1).

Recent studies have highlighted that RCC cells undergo metabolic reprogramming—a process whereby tumor cells restructure their metabolic pathways to meet the demands of uncontrolled proliferation and malignant progression. This reprogramming involves significant alterations in key metabolic pathways, including glucose, lipid, and amino acid metabolism (Jonasch et al., 2021b). Such metabolic dysregulation is often driven by aberrant expression of critical regulatory molecules and enzymes, which are closely associated with tumor progression, therapy resistance, and patient prognosis. These molecules show great promise as diagnostic and prognostic biomarkers as well as potential therapeutic targets.

This review aims to comprehensively summarize the major features of metabolic reprogramming in RCC, examine recent advances in identifying metabolism-related biomarkers, and explore their clinical implications in diagnosis, prognostic assessment, and therapeutic decision-making. Ultimately, we seek to provide novel theoretical insights and future research directions for the precision diagnosis and treatment of RCC.

## 2 Brief overview of metabolic reprogramming

### 2.1 Metabolic reprogramming in RCC

Recent studies on metabolic dysregulation in RCC have revealed how tumor cells undergo metabolic reprogramming to sustain energy production and adapt to the tumor microenvironment. In ccRCC, the characteristic translucent cytoplasm, along with prominent accumulation of lipids and glycogen, reflects this profound metabolic shift. Analysis of nearly 500 ccRCC samples by The Cancer Genome Atlas (TCGA) further confirmed a marked downregulation of the tricarboxylic acid (TCA) cycle, accompanied by significant upregulation of the pentose phosphate pathway (PPP), fatty acid synthesis, and glutamine transport (The Cancer Genome Atlas Research Network, 2013). This metabolic reprogramming not only supports rapid tumor cell proliferation and growth but also correlates strongly with poor prognosis and reduced patient survival.

At the molecular level, metabolic reprogramming in ccRCC is characterized by enhanced aerobic glycolysis (the Warburg effect), increased PPP activity, suppression of the TCA cycle and oxidative phosphorylation (OXPHOS), elevated fatty acid synthesis, and reduced fatty acid β-oxidation (FAO). Additionally, dysregulated cholesterol metabolism, upregulated glutamine metabolism, elevated glutathione/glutathione disulfide (GSH/GSSG) pathway activity, and aberrant tryptophan and arginine metabolism are frequently observed. Key regulatory genes involved in these processes include VHL, PTEN, Akt, mTOR, TSC1/2, Myc, PBRM1, BAP1, SETD2, KDM5C, and TP53 (Linehan et al., 2010; Haake et al., 2016; Yang et al., 2014; Zheng et al., 2021; Gossage et al., 2015; Turajlic et al., 2018). And genes associated with hereditary RCC include ELOC, MET, TSC, FLCN, MITF, TFE3 (Coffey and Simon, 2024).

Among them, *VHL* inactivation represents one of the most common molecular features of ccRCC. Loss of *VHL* function leads to the stabilization and accumulation of HIF-1α and HIF-2α, creating a state of “pseudohypoxia” that drives the expression of genes involved in tumor growth, angiogenesis, metastasis, and glucose metabolism (Jaakkola et al., 2001). Studies have suggested that while HIF-1α may exert tumor-suppressive effects in ccRCC, HIF-2α is predominantly oncogenic (Hanahan and Weinberg, 2011).

Moreover, frequent mutations in components of the PI3K-Akt-mTOR signaling pathway—such as PTEN, TSC1/2, and PIK3CA—further promote metabolic reprogramming (Voss et al., 2014; Dibble and Cantley, 2015; Inoki et al., 2002; Wee et al., 2008). Notably, hyperactivation of mTORC1 enhances metabolic adaptation by suppressing 4E-BP1 and increasing HIF expression (Düvel et al., 2010; Toschi et al., 2008). In addition, *Myc*, a well-known proto-oncogenic transcription factor, upregulates several genes implicated in tumorigenesis and plays a pivotal role in ccRCC metabolic reprogramming, particularly by promoting glutamine metabolism and fatty acid synthesis (Gordan et al., 2007).

Importantly, large-scale genomic sequencing efforts have revealed that mutations in PBRM1, BAP1, SETD2, KDM5C, and TP53—many located on chromosome 3p, often co-deleted with VHL—are among the most frequent genetic alterations in ccRCC. PBRM1, a subunit of the SWI/SNF chromatin-remodeling complex, is mutated in ∼40% of cases and modulates transcriptional regulation, immune microenvironment, and response to immune checkpoint blockade (Wu et al., 2023d). BAP1, a deubiquitinase, is mutated in 10%–15% of cases, correlating with aggressive histology, poor prognosis, and metabolic dysregulation (Wu et al., 2023d). SETD2, the sole histone H3K36 trimethyltransferase, is inactivated in ∼12% of cases, impairing DNA repair and genome stability (Gossage et al., 2015). KDM5C, a histone demethylase frequently mutated in ccRCC, regulates chromatin accessibility and metabolic gene expression, linking epigenetics to tumor progression (Zheng et al., 2021). TP53, although mutated less frequently in ccRCC compared to other cancers, is associated with higher tumor grade, treatment resistance, and adverse outcomes when present (Turajlic et al., 2018).

In hereditary RCC, particularly papillary renal cell carcinoma (pRCC), tumorigenesis is influenced by multiple genetic and metabolic drivers, with marked heterogeneity observed between type 1 and type 2 subtypes. In hereditary papillary renal carcinoma (hpRCC), germline gain-of-function mutations in the proto-oncogene MET confer a 100% lifetime risk of developing type 1 pRCC, and Met alterations are also frequent in sporadic cases, where 15%–20% of tumors harbor activating mutations and up to 90% show chromosomal copy number gains (Maher, 2018; Linehan et al., 2016; Yin et al., 2015). PTEN loss in Cowden syndrome and TSC1/2 mutations in tuberous sclerosis complex are additional drivers that converge on hyperactivation of the PI3K–AKT–mTOR signaling pathway, thereby promoting tumorigenesis (Maher, 2018; Kim et al., 2020; Yang et al., 2014). Moreover, hereditary and sporadic forms of FH and SDH deficiency represent key drivers of type 2 pRCC, linking dysregulated tricarboxylic acid (TCA) cycle activity with accumulation of oncometabolites such as fumarate and succinate (Maher, 2018; Vanharanta et al., 2004; Lehtonen et al., 2004). Translocations of TFE3 and TFEB further contribute to oncogenesis in a subset of type 2 tumors by driving aberrant mTOR signaling (Moch et al., 2022). At the metabolic level, pRCC exhibits impaired oxidative metabolism and enhanced glycolysis, with single-cell analyses revealing that disease progression is associated with profound metabolic dysfunction mediated by upregulation of LDHA and downregulation of FH, highlighting their importance as functional drivers of tumor aggressiveness (Wang et al., 2022b).

### 2.2 Glucose metabolism

**FIGURE 2:**
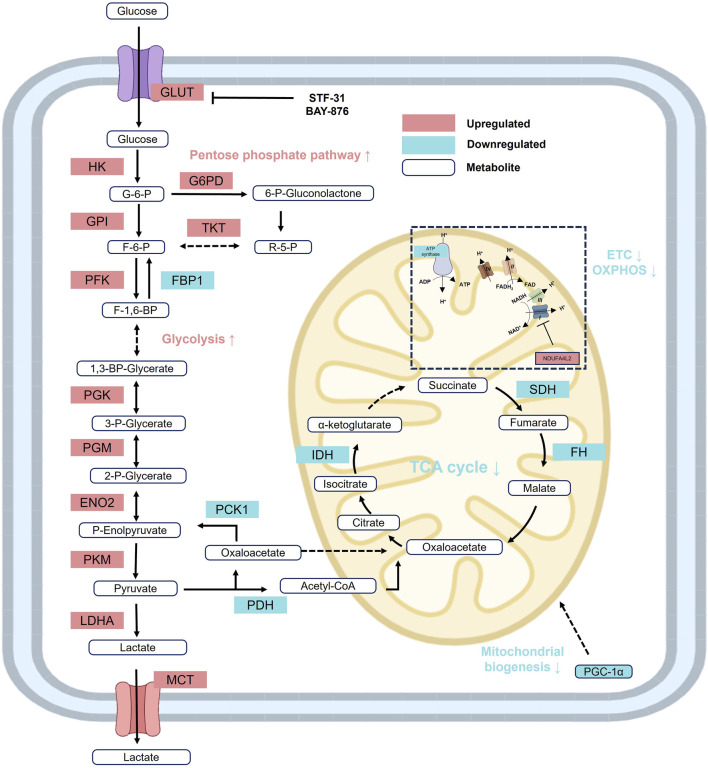
Reprogramming of glucose metabolism in RCC. In RCC, glycolysis and the PPP are markedly upregulated, while gluconeogenesis, the TCA cycle, and oxidative phosphorylation (OXPHOS) are significantly suppressed, reflecting a classical Warburg effect or aerobic glycolysis metabolic pattern. Red: increased activity; Blue: decreased activity. Abbreviations: ENO, enolase; FBP1, fructose-1-bisphophatase; FH, fumarase; F-1,6-BP, fructose 1,6-bisphosphate; F-6-P, fructose 6-phosphate; GPI, glucose-6-phosphate isomerase; HK, hexokinase; IDH, isocitrate dehydrogenase; LDH, lactate dehydrogenase; PDH, pyruvate dehydrogenase; P-Enolpyruvate, phosphoenolpyruvate; PFK, phosphofructokinase; PGK, phosphoglycerate kinase; PGM, phosphoglycerate mutase; PGC-1α, peroxisome proliferator-activated receptor gamma coactivator 1-α; PKM2, phosphoglycerate mutase; R-5-P, ribose-5-phosphate; SDH, succinate dehydrogenase; TKT, transketolase; 1,3-BP-Glycerate, 1,3-bisphospho-D-glycerate; GLUT, glucose transporter; 2-P-Glycerate, 2-phosphoglycerate; 3-P-Glycerate, 3-phosphoglycerate; 6-P-Gluconolactone, 6-phosphoglucono-d-lactone; G6PD; glucose- 6-phosphate dehydrogenase; MCT, Monocarboxylate transporter.

Glucose metabolism undergoes significant reprogramming in RCC, primarily involving cytoplasmic glycolysis, the pentose phosphate pathway (PPP), and mitochondrial TCA cycle and OXPHOS (Figure 2). In RCC cells, glycolysis and the PPP are markedly upregulated, whereas the TCA cycle and OXPHOS are significantly suppressed—metabolic features that constitute the classical Warburg effect or aerobic glycolysis (Vander Heiden et al., 2009). Concurrently, RCC cells exhibit enhanced dependence on glycolysis by downregulating key gluconeogenic enzymes, including glucose-6-phosphatase (G6PC), fructose-1,6-bisphosphatase 1 (FBP1), and phosphoenolpyruvate carboxykinase 1 (PCK1) (Xu et al., 2020c; Li et al., 2014; Shi et al., 2020). Recent studies further demonstrate that VHL loss not only enhances glycolytic activity in cancer cells but also profoundly remodels the tumor microenvironment (TME). Specifically, tumor-associated macrophages (TAMs) in VHL-deficient tumors display increased glucose uptake, phagocytic activity, and proinflammatory transcriptional signatures, indicating that glycolysis-driven reprogramming extends beyond tumor cells to immune infiltrates within the TME (Wolf et al., 2024).

Although substantial glycogen accumulation is observed in RCC cells, regulation of glycogen metabolism appears to have minimal impact on tumor growth, suggesting that glycogen storage may be a secondary consequence of abnormal HIF activity rather than a direct driver of malignancy (Xie et al., 2021). Meanwhile, upregulation of the PPP supplies essential intermediates such as NADPH and ribose-5-phosphate, which are required for lipid biosynthesis and nucleotide production, thereby supporting the rapid proliferation of tumor cells (Wanders et al., 2001). Importantly, the glycolytic bias in the TME also contributes to immune dysfunction, as T cells in VHL-deficient tumors show reduced effector cytokine production and diminished response to PD-1 blockade, highlighting metabolic competition between tumor and immune cells as a key determinant of therapeutic resistance (Wolf et al., 2024).

The downregulation of OXPHOS is also closely linked to increased tumor aggressiveness (Simonnet et al., 2002). HIF-1α directly suppresses the expression and activity of mitochondrial electron transport chain complexes, thereby promoting a metabolic shift toward glycolysis for ATP production (Lunt and Vander Heiden, 2011). This reprogrammed metabolic landscape not only provides a foundation for RCC progression but also highlights potential metabolic vulnerabilities that may serve as novel therapeutic targets.

### 2.3 Lipid metabolism

**FIGURE 3:**
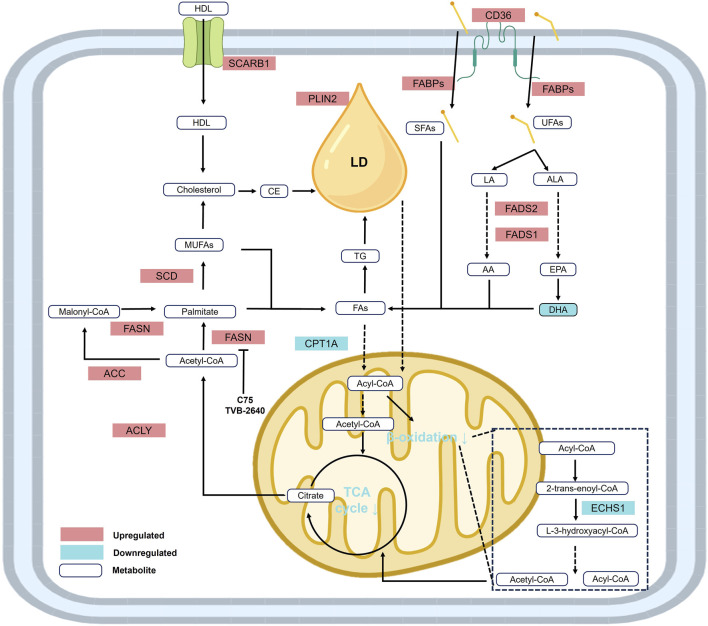
Reprogramming of lipid metabolism in RCC. Under the context of lipid metabolic reprogramming, fatty acid and lipid synthesis are significantly upregulated, while fatty acid β-oxidation is downregulated. In addition, elevated levels of metabolites and enzymes involved in cholesterol biosynthesis contribute to the accumulation of cholesterol, fatty acids, triglycerides, unsaturated fatty acids (UFAs), and lipid droplets, establishing the distinct histological phenotype of renal cell carcinoma. Abbreviations: AA, arachidonic acid; ACC, acetyl-CoA carboxylase; ACLY, ATP-citrate lyase; ALA, α-linolenic acid; CPT1A, carnitine palmitoyltransferase 1-A; CE, cholesteryl ester; DHA, docosahexaenoic acid; ECHS1, enoyl-CoA hydratase, short chain 1; EPA, eicosapentaenoic acid; FABPs, fatty acid binding proteins; FADS1, fatty acid desaturase 1; FADS2, fatty acid desaturase 2; FASN, fatty acid synthase; FAs, fatty acids; HDL, high-density lipoprotein; LA, linoleic acid; LD, lipid droplet; MUFAs, monounsaturated fatty acids; PLIN2, perilipin 2; SCARB1, scavenger receptor B1; SCD, stearoyl-CoA desaturase; SFAs, saturated fatty acids; UFAs, unsaturated fatty acids.

Lipid metabolic reprogramming in ccRCC is characterized by upregulated lipid synthesis and storage, alongside suppressed lipid utilization and oxidation (Ganti et al., 2012a). This imbalance results in the accumulation of cholesterol (Gebhard et al., 1987), fatty acids (Hakimi et al., 2016), triglycerides (Heravi et al., 2022), phospholipids, and polyunsaturated fatty acids (PUFAs) (Figure 3). The dysregulation of multiple metabolic pathways synergistically promotes membrane biosynthesis, cell proliferation, and drives tumor progression (Wettersten et al., 2017).

Enhanced fatty acid synthesis is a hallmark of ccRCC, with key enzymes such as ATP citrate lyase (ACLY) and fatty acid synthase (FASN) significantly upregulated under the activation of HIF and mTOR signaling. These enzymes catalyze the conversion of acetyl-CoA into saturated fatty acids, providing lipid precursors essential for membrane formation and energy storage in the form of lipid droplets (Taylor and Scholz, 2022).

Conversely, fatty acid oxidation (FAO) is suppressed through the downregulation of carnitine palmitoyltransferase 1A (CPT1A) and HIF-2α-mediated peroxisomal dysfunction, limiting lipid catabolism for energy production. This shift reduces reactive oxygen species (ROS) generation to support cell survival, while rerouting metabolic flux toward synthetic pathways, thereby accelerating lipid accumulation (Du et al., 2017).

These metabolic features are governed by the global regulation of the VHL-HIF axis and redox homeostasis, collectively forming a “lipid addiction” phenotype that contributes to drug resistance and immune evasion. Targeting key nodes in lipid metabolism—such as inhibiting FASN or ACLY, or restoring FAO—disrupts lipid homeostasis and represents a promising therapeutic strategy for ccRCC (Xiong et al., 2021).

### 2.4 Amino acid metabolism

**FIGURE 4:**
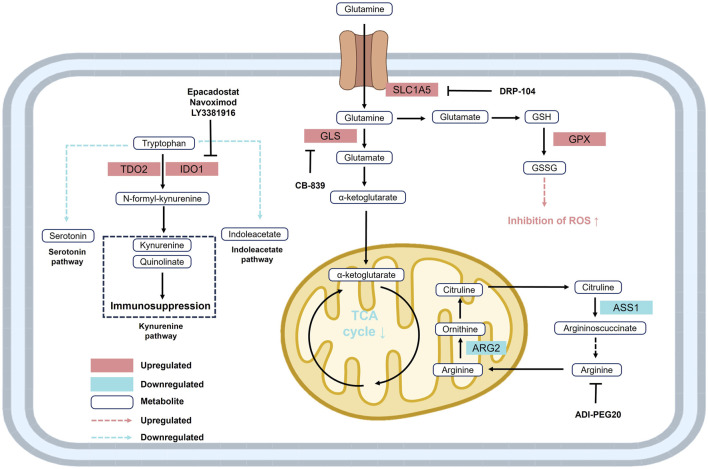
Reprogramming of glutamine, tryptophan, and arginine metabolism in RCC. Upregulation of SLC1A5 and GLS enhances glutamine metabolism, supporting the TCA cycle and redox balance. Elevated TDO2 and IDO drive tryptophan conversion to kynurenine, promoting immunosuppression. Increased ARG2 and decreased ASS1 lead to arginine auxotrophy. These alterations facilitate ccRCC cell survival and immune evasion. Abbreviations: ARG2, arginase 2; ASS1, argininosuccinate synthase 1; GLS, glutaminase; GPX, glutathione peroxidase; GSH, glutathione; GSSG, reduced glutathione; IDO, indoleamine 2,3-dioxygenase; SLC1A5, solute carrier family 1 member 5; TDO2, tryptophan 2,3-dioxygenase 2.

Glutamine metabolism plays a pivotal role in ccRCC. Glutamine is primarily transported into the cell via the solute carrier SLC1A5 and is subsequently converted to glutamate by glutaminase (GLS). Glutamate is then metabolized into α-ketoglutarate (α-KG), which enters the TCA cycle or is redirected through reductive carboxylation to generate isocitrate for lipid biosynthesis (Hassanein et al., 2013). ccRCC cells frequently exhibit marked glutamine addiction, and either GLS inhibition or glutamine deprivation significantly reduces tumor cell viability (Abu Aboud et al., 2017). Moreover, glutamine serves as a precursor for the GSH. Elevated levels of glutamine, glutamate, and the GSH/GSSG ratio are commonly observed in ccRCC cells, contributing to ROS detoxification and redox homeostasis through glutathione peroxidase 1 (GPX1)-mediated mechanisms (Hakimi et al., 2016; Figure 4).

Arginine metabolism is also distinctly dysregulated in ccRCC. Downregulation of argininosuccinate synthetase 1 (ASS1) enhances the tumor’s dependence on extracellular arginine (Rabinovich et al., 2015). Loss or suppression of ASS1 expression is commonly observed in various malignancies and is associated with chemoresistance and poor prognosis, rendering tumors more sensitive to arginine-depleting therapies such as ADI-PEG20 (Delage et al., 2010). Notably, restoration of ASS1 expression has been shown to significantly inhibit tumor growth, highlighting the arginine metabolic pathway as a promising therapeutic target (Yoon et al., 2007; Figure 4).

Tryptophan metabolism is similarly altered in ccRCC, primarily through activation of the kynurenine (KN) pathway mediated by indoleamine 2,3-dioxygenase (IDO) (Wettersten et al., 2017). IDO overexpression not only depletes intracellular tryptophan and facilitates immune evasion, but also produces immunosuppressive metabolites such as kynurenine and quinolinic acid, which inhibit effector T cell function and promote tumor invasiveness (Fiore and Murray, 2021). Although IDO inhibitors have entered clinical trials in combination therapies, their efficacy in ccRCC remains limited, indicating that further investigation is needed to elucidate the precise mechanisms underlying tryptophan metabolism (Li et al., 2019; Figure 4).

## 3 Biomarkers in metabolic reprogramming

### 3.1 Biomarkers in glucose metabolism

#### 3.1.1 Glycolysis

##### 3.1.1.1 GLUT (glucose transporter)

In the field of RCC research, the glucose transporter (GLUT) family has garnered increasing attention for its critical roles in tumor metabolic reprogramming, cancer progression, immune evasion, and therapeutic resistance mechanisms. Among this family, GLUT1 is one of the most extensively studied members, with its expression significantly upregulated in various malignancies, including ccRCC (Singer et al., 2011). Notably, in ccRCC VHL gene deficiency, HIF-1α is highly activated, markedly enhancing GLUT1 expression (Ivan and Huang, 2014). In addition, oncogenic Ras and NF-κB signaling pathways have been shown to upregulate the expression of GLUT1 and GLUT3, respectively, thereby increasing glucose uptake efficiency in renal cancer cells (Zha et al., 2015). Recent studies have revealed that S100A2 interacts with the transcription factor HNF1A to cooperatively activate GLUT2 expression, which significantly enhances glucose uptake and glycolytic activity, ultimately promoting ccRCC cell migration and invasion. This newly identified S100A2-HNF1A-GLUT2 axis offers a potential therapeutic target for advanced ccRCC (Deng et al., 2025).

At the diagnostic and therapeutic level, 18F-FDG PET imaging, a non-invasive detection modality that leverages glucose analog probes (FDG) closely associated with GLUT1-mediated glucose uptake, has been demonstrated to effectively assess the response of ccRCC patients to targeted therapies such as PI3K, AKT, and mTOR inhibitors. This technology serves as both a pharmacodynamic and prognostic biomarker, providing critical insights for treatment monitoring and personalized therapeutic strategies in patients with advanced renal cancer (Mizuno et al., 2015; Ma et al., 2009; Nakaigawa et al., 2016; Table 1). However, its clinical application faces several important limitations. First, low-grade or certain histological subtypes such as chromophobe RCC exhibit insufficient FDG uptake, resulting in reduced sensitivity and potential underestimation of malignancy (Takahashi et al., 2015). Second, active inflammatory or infectious processes can lead to false-positive findings due to FDG accumulation in activated immune cells, which may confound interpretation without correlation to clinical context or complementary imaging (Chang et al., 2006). Third, the inherently high metabolic background of renal parenchyma and FDG excretion into the urinary tract complicate differentiation between tumor lesions and normal structures (Takahashi et al., 2015). Fourth, the limited spatial resolution of PET leads to partial-volume effects, particularly for lesions smaller than 6–8 mm, which may cause underestimation of SUV or complete omission (Purohit et al., 2014). Fifth, heterogeneity in GLUT1 expression across RCC subtypes and the contribution of alternative metabolic pathways such as GLUT5 or glucose-6-phosphatase (G6Pase) further affect FDG kinetics and imaging accuracy (Takahashi et al., 2015). Finally, while SUVmax has been associated with poor recurrence-free survival, its prognostic power is improved when combined with systemic inflammatory markers such as C-reactive protein (CRP); however, CRP is non-specific and may be influenced by infection or comorbidities. These limitations highlight the need for integrating FDG PET with anatomical imaging modalities, multiparametric PET/MRI, radiomics, and clinicopathological parameters to improve reliability and accuracy in the individualized management of ccRCC.

**TABLE 1:** Functions and clinical biomarker value of molecules involved in glucose metabolism reprogramming in RCC.

Molecule	Function	Biomarker role or clinical value	References
GLUT	Mediates glucose transmembrane transport.	GLUT1 expression is upregulated and used in PET imaging to evaluate targeted therapy response; influenced by VHL loss and HIF-1α activation, indicating poor prognosis. GLUT2 promotes tumor migration and invasion.	Mizuno et al. (2015), Ma et al. (2009), Ivan and Huang (2014), Deng et al. (2025)
HK	Catalyzes the phosphorylation of glucose to glucose-6-phosphate, initiating glycolysis.	High HK2 expression is associated with advanced tumor stage, lymph node metastasis, and poor survival, serving as an independent prognostic factor. HK3 is co-expressed with multiple immunosuppressive molecules, suggesting a role in immune regulation.	Yin et al. (2015), Xu et al. (2021)
PFK1	Rate-limiting enzyme of glycolysis that catalyzes the conversion of fructose-6-phosphate.	High expression is closely associated with poor prognosis.	Wang et al. (2016b)
PFKFB	Regulates fructose-2,6-bisphosphate synthesis and PFK1 activity.	PFKFB3 overexpression correlates with advanced TNM stage. PFKFB4 promotes PPP metabolism and tumor invasion, and its inhibition reverses sunitinib resistance.	Li et al. (2022), Feng et al. (2021)
PGK1	Catalyzes the conversion of 1,3-bisphosphoglycerate to 3-phosphoglycerate, generating ATP.	Overexpression enhances glycolysis and activates the CXCR4/AKT/ERK pathway, promoting sorafenib resistance and correlating with poor prognosis.	Schönenberger et al. (2016), He et al. (2022)
PGAM1	Catalyzes the conversion of 3-phosphoglycerate to 2-phosphoglycerate.	Overexpression is linked to macrophage infiltration. Inactivation increases drug sensitivity, making it a potential target for metabolic intervention.	, Wen et al. (2023)
ENO2	Catalyzes the conversion of 2-phosphoglycerate to phosphoenolpyruvate.	Promotes tumor proliferation and migration; strongly associated with poor prognosis. High expression correlates with higher tumor grade and shorter survival, serving as a diagnostic and prognostic biomarker with therapeutic potential.	Shi et al. (2023), Chen et al. (2023), Li et al. (2024b)
LDHA	Converts pyruvate to lactate, maintaining the Warburg effect.	Its overexpression is linked to tumor size, clinical stage, EMT, and metastasis. Also involved in drug resistance and represents a potential target for metabolic and epigenetic therapy.	Coffey and Simon (2024), Zhao et al. (2017)
MCT	Transports monocarboxylates such as lactate and pyruvate, maintaining intracellular and extracellular lactate balance.	Expression correlates with tumor size and TNM stage; valuable for targeted therapy and risk stratification.	Fisel et al. (2013), de Carvalho et al. (2021)
SDH	Mitochondrial enzyme complex catalyzing the conversion of succinate to fumarate, linking the TCA cycle and electron transport chain.	SDHB is commonly downregulated in ccRCC, leading to succinate accumulation, HIF activation, CIMP, and EMT. SDHB loss on IHC is the diagnostic gold standard for SDH-deficient RCC and indicates poor prognosis and high metastatic risk.	Sciacovelli et al. (2016), Fuchs et al. (2022)
FH	Catalyzes the conversion of fumarate to malate, preserving TCA cycle integrity.	FH deficiency causes fumarate accumulation, inducing pseudohypoxia, CIMP, and EMT, indicative of high tumor aggressiveness. FH-deficient tumors are sensitive to PARP and purine metabolism inhibitors. Plasma markers such as SAICAR/succinylcysteine may assist in early detection and recurrence monitoring.	Sciacovelli et al. (2016), Wilde et al. (2023), Zheng et al. (2023)
IDH	Catalyzes the conversion of isocitrate to α-ketoglutarate, regulating energy metabolism and redox balance.	Downregulation or mutation of IDH mediates reductive carboxylation of glutamine to lipid, promoting tumor growth. Mutant IDH-derived metabolites possess diagnostic and therapeutic monitoring potential.	Shim et al. (2014), Lee et al. (2024)
PGC-1α	A transcriptional coactivator of mitochondrial biogenesis and OXPHOS, enhancing OCR and ATP production.	Downregulated expression is associated with mitochondrial dysfunction, glycolytic phenotype, and poor prognosis.	LaGory et al. (2015), Koh et al. (2018)
ATP Synthase Subunits and Assembly Factor	Structural subunits and assembly factors of ATP synthase, essential for ATP production and mitochondrial respiration.	Generally downregulated; negatively correlated with stage, grade, and overall survival. Low ATP5A1 promotes proliferation, migration, and invasion, and activates the Wnt/β-catenin pathway.	Brüggemann et al. (2017), Zhou et al. (2025)
NDUFS1	A subunit of ETC complex I, involved in electron transfer and mitochondrial respiratory chain function.	Loss of expression impairs complex I function and decreases OCR, strongly associated with poor survival in ccRCC; reflects OXPHOS dysfunction.	Bezwada et al. (2023), Ellinger et al. (2017)
NDUFA4L2	A HIF-1α-regulated inhibitor of complex I, suppressing OXPHOS and reducing ROS production.	Highly expressed in tumors, facilitates hypoxic adaptation and invasion, associated with tumor progression and poor prognosis. Co-upregulated with SHMT2; a potential therapeutic target.	Minton et al. (2016), Liu et al. (2016), Wang et al. (2020a)
UQCRC1	Core subunit of ETC complex III, regulates electron transport and ATP synthesis efficiency.	Low expression correlates with reduced survival; promoter hypermethylation suggests epigenetic regulation, serving as a prognostic and subclassification marker.	Ellinger et al. (2016)
UQCRH	Hinge protein of ETC complex III, facilitates cytochrome c electron transfer and mitochondrial apoptosis.	Downregulated expression correlates with tumor progression and malignancy; its overexpression restores mitochondrial function and promotes apoptosis, acting as a tumor suppressor and functional biomarker.	Luo et al. (2020)
G6PD	Rate-limiting enzyme in PPP, generates NADPH and ribose-5-phosphate, maintaining redox balance and supporting nucleotide synthesis.	Overexpression correlates with tumor aggressiveness and poor prognosis; an independent predictor of poor postsurgical survival. Positively associated with immune infiltration and immunoregulation.	Zhang et al. (2022), Liu et al. (2023)
G6PI	Glycolytic enzyme catalyzing G-6-P to F-6-P conversion; also acts as an autocrine motility factor.	Significantly overexpressed and co-localized with AMFR. High expression shortens CSS and PFS, suggesting a key role in tumor invasion and progression.	Lucarelli et al. (2015b)
TKT	Key enzyme of the non-oxidative PPP branch, mediates interconversion of glycolytic and pentose intermediates.	High expression correlates with advanced stage, metastasis, and poor survival. Induced by miR-146a-5p, driving metabolic reprogramming and invasion, with prognostic and classification value.	Ricketts et al. (2018), Bogusławska et al. (2019)
PCK1	Rate-limiting enzyme of gluconeogenesis, converts oxaloacetate to phosphoenolpyruvate, counteracting glycolysis.	Downregulation correlates with elevated LDHA expression and enhanced^18^F-FDG uptake. Stabilization of LDHA is suppressed by PCK1, indicating its role as a metabolic modulator and tumor suppressive marker.	Shi et al. (2020)
G6PC	Catalyzes the final step of gluconeogenesis, converting G6P to glucose for systemic release.	Reduced expression is strongly associated with shorter overall survival, indicating high-risk, aggressive ccRCC.	Xu et al. (2020c)
FBP1	Catalyzes F-1,6-BP to F-6-P conversion, antagonizing glycolysis and maintaining metabolic balance.	Downregulation enhances Warburg effect and tumor invasiveness, indicating poor prognosis. Ectopic expression inhibits tumor growth, making it a potential metabolic therapy target.	Ju et al. (2022)

##### 3.1.1.2 HK (hexokinase)

The hexokinase (HK) family consists of HK1, HK2, HK3, and HK4 (also known as glucokinase, GCK). These enzymes catalyze the conversion of glucose to glucose-6-phosphate (G6P), the first rate-limiting step of glycolysis, thereby playing a fundamental role in cellular energy metabolism and glucose utilization. Among them, HK2 is significantly upregulated in RCC tissues and is strongly associated with advanced tumor stage, lymph node metastasis, and poor overall survival. Moreover, HK2 has been identified as an independent prognostic risk factor and is positively correlated with immune cell infiltration, suggesting a critical role in RCC progression and immune regulation (Yin et al., 2015) Simultaneously, HK3 and GLUT1 are upregulated in ccRCC, and their expression is linked to an immunosuppressive tumor phenotype. Specifically, HK3 expression positively correlates with immune checkpoint molecules such as IDO1, CTLA-4, PD-1, PD-L1, Siglec-15, and PD-L2, implying that the glycolytic pathway plays a key role in modulating immune cell function (Xu et al., 2021). Beyond its canonical role in phosphorylating glucose, HK2 also facilitates the transfer of phosphate groups from ATP to the E1α subunit of pyruvate dehydrogenase (PDHA1), markedly increasing phosphorylation at the Ser293 residue. This modification inhibits PDH complex activity and promotes the persistence of the Warburg effect. Notably, HK2 overexpression is closely associated with elevated PDHA1 phosphorylation and disease progression in ccRCC, further underscoring its central role in metabolic reprogramming (Luo et al., 2019; Table 1).

##### 3.1.1.3 PFK1/PFKFB/PGK1/PGAM1 enzyme cluster

Several key glycolytic enzymes—including phosphofructokinase (PFK), phosphoglycerate kinase 1 (PGK1), and phosphoglycerate mutase (PGAM)—are upregulated to varying degrees in RCC, and their elevated expression levels are consistently associated with poor patient prognosis.

Phosphofructokinase-1 (PFK1), a rate-limiting enzyme in glycolysis, is markedly upregulated in ccRCC, particularly the platelet isoform (PFK-P), which is specifically overexpressed in ccRCC. Knockdown of PFK-P has been shown to induce apoptosis and cell cycle arrest in ccRCC cells, significantly inhibiting their proliferative capacity *in vitro* (Wang et al., 2016b). In addition, PFKFB family members, which functionally regulate PFK1 activity—namely PFKFB3 and PFKFB4—are also aberrantly overexpressed in RCC. PFKFB3 expression in RCC tissues and cell lines correlates with advanced TNM stage and serves as a robust prognostic biomarker. Its silencing significantly reduces glycolytic activity, inhibits cell proliferation and G1/S phase progression, and delays tumor growth *in vivo*, highlighting its potential as a therapeutic target in RCC (Li et al., 2022). Similarly, PFKFB4 overexpression is strongly associated with increased invasiveness of ccRCC and enhanced PPP activity. Mechanistically, PFKFB4 modulates FBP1 through phosphorylation of NCOA3, forming a negative feedback loop that maintains tumor metabolic balance. Notably, inhibition of PFKFB4 has been shown to reverse ccRCC resistance to the antiangiogenic agent sunitinib, underscoring its therapeutic promise (Feng et al., 2021; Table 1).

Phosphoglycerate kinase 1 (PGK1), another glycolytic enzyme upregulated in RCC, is significantly overexpressed in both ccRCC tumor tissues and patient serum, and is associated with poor prognosis (Schönenberger et al., 2016). Studies have demonstrated that PGK1 overexpression in ccRCC is accompanied by upregulation of other glycolytic enzymes and the chemokine receptor CXCR4, thereby enhancing glycolytic flux and activating CXCR4-mediated AKT and ERK signaling pathways. Furthermore, PGK1 contributes to resistance against the tyrosine kinase inhibitor sorafenib through activation of the CXCR4–ERK axis (He et al., 2022; Table 1).

Phosphoglycerate mutase 1 (PGAM1) is also broadly overexpressed in RCC and several other cancer types. Using single-cell and spatial transcriptomic analyses, Wen et al. revealed that PGAM1 upregulation in ccRCC is closely associated with immune cell infiltration, particularly macrophage enrichment. Moreover, PGAM1 inactivation has been linked to increased sensitivity to specific small-molecule drugs (Wen et al., 2023). These findings suggest that targeting PGAM1 and related glycolytic enzymes could represent a promising strategy to improve therapeutic outcomes in RCC (Table 1).

##### 3.1.1.4 ENO2 (enolase 2)

ENO2 (enolase 2, also known as neuron-specific enolase) is a key glycolytic enzyme that catalyzes the conversion of 2-phosphoglycerate to phosphoenolpyruvate (PEP), and its upregulation in various malignancies has garnered increasing attention in recent years. In both ccRCC and papillary RCC (pRCC), ENO2 is markedly overexpressed and is strongly associated with poor clinical outcomes (Shi et al., 2023; Chen et al., 2023). Mechanistically, ENO2 has been identified as a downstream target of HIF-1α. Studies have shown that HIF-1α transcriptionally activates ENO2 expression in renal cancer cells, thereby enhancing glycolytic activity, promoting glucose metabolism, and further supporting ccRCC proliferation and invasiveness—a metabolic hallmark closely aligned with the classical Warburg effect (Shi et al., 2023). At the cellular functional level, elevated ENO2 expression significantly promotes the migration, invasion, and proliferation of ccRCC cells. Given its pronounced oncogenic role in both ccRCC and pRCC, as well as its negative prognostic implications, multiple studies have proposed ENO2 as a diagnostic and prognostic biomarker with clear therapeutic potential (Shi et al., 2023; Li et al., 2024b). High ENO2 expression is not only indicative of higher tumor grade and shorter survival but also suggests that therapeutic targeting of ENO2 and its associated signaling pathways may offer a novel strategy to improve RCC treatment outcomes (Shi et al., 2023; Chen et al., 2023; Li et al., 2024b; Table 1).

##### 3.1.1.5 LDHA (lactate dehydrogenase A)

LDHA (lactate dehydrogenase A) is a key enzyme at the end of glycolysis that catalyzes the conversion of pyruvate to lactate while regenerating NAD^+^, thereby sustaining glycolytic flux. Single-cell sequencing analyses in pRCC have identified LDHA as one of the most significantly upregulated genes, with this metabolic alteration strongly associated with disease progression to advanced stages and poor survival outcomes (Coffey and Simon, 2024). In ccRCC, LDHA expression is significantly positively correlated with tumor size, clinical stage, and histological grade. Conversely, its expression is negatively correlated with both disease-free survival and overall survival.

Mechanistically, FK506-binding protein 10 (FKBP10) has been shown to directly interact with LDHA via its C-terminal domain in ccRCC, enhancing LDHA phosphorylation at the Y10 site. This modification promotes the Warburg effect and the accumulation of histone lactylation, thereby driving tumor proliferation and metastasis. Moreover, HIFα negatively regulates FKBP10 expression, and inhibition of FKBP10 has been found to enhance the antitumor efficacy of the HIF-2α inhibitor PT2385 (Liu et al., 2024).

Beyond its role in metabolic regulation, LDHA overexpression is closely linked to increased tumor invasiveness, migration, and epithelial–mesenchymal transition (EMT). In RCC tissues, LDHA expression is markedly elevated and strongly associated with poor postoperative survival. Notably, LDHA levels correlate positively with the EMT marker N-cadherin and negatively with E-cadherin, suggesting that LDHA may facilitate RCC cell invasion and metastasis by modulating the EMT process (Zhao et al., 2017; Table 1).

##### 3.1.1.6 MCT (monocarboxylate transporters)

Monocarboxylate transporters (MCTs) are a family of transmembrane proteins that mediate the transport of monocarboxylate molecules, such as lactate and pyruvate, across the cell membrane. In ccRCC, the expression levels of MCT1 and MCT4 are significantly upregulated and are strongly associated with tumor progression and poor patient prognosis (Fisel et al., 2013). Through immunohistochemical analysis of specimens from patients receiving VEGFR-targeted therapy, Cao et al. demonstrated that high expression of MCT1 and MCT4 serves as an independent prognostic marker for reduced progression-free survival (PFS), indicating their potential value in targeted therapy and risk stratification (de Carvalho et al., 2021). Additionally, Paulo et al., using tissue microarray analysis from 207 ccRCC patients who underwent nephrectomy, found that MCT1 expression was significantly correlated with classical prognostic indicators such as tumor size and TNM stage. Multivariate analysis further confirmed MCT1 as an independent prognostic factor for cancer-specific survival in ccRCC (de Carvalho et al., 2021; Table 1).

Collectively, glycolytic reprogramming in RCC is orchestrated through the coordinated actions of multiple key regulators. GLUT1, GLUT2, and GLUT3 ensure sufficient glucose influx, while HK2 and HK3 catalyze its initial phosphorylation and simultaneously reprogram mitochondrial pyruvate metabolism by suppressing PDH activity, thereby reinforcing glycolytic dependence. PFKFB3 and PFKFB4 sustain high glycolytic flux by activating PFK1 and redirecting intermediates to the pentose phosphate pathway, which supports redox balance and tumor survival. Downstream, PGK1 and PGAM1 not only accelerate glycolytic throughput but also couple metabolic activity with oncogenic signaling and immune microenvironment remodeling. ENO2, under HIF-1α regulation, amplifies the Warburg effect and further drives tumor proliferation and invasion. At the terminal stage, LDHA ensures the continuous conversion of pyruvate to lactate and promotes histone lactylation, thereby linking energy metabolism to epigenetic regulation. These enzymes collectively form a synergistic metabolic circuit that facilitates tumor growth, immune evasion, EMT induction, and therapeutic resistance, highlighting the importance of targeting glycolysis as an integrated network rather than as isolated nodes in the treatment of RCC.

#### 3.1.2 TCA cycle

##### 3.1.2.1 SDH (succinate dehydrogenase)

Succinate dehydrogenase (SDH) is a mitochondrial enzyme complex that bridges the TCA cycle and the electron transport chain (ETC), and has emerged as a potential biomarker in renal cell carcinoma (RCC). Comprising four subunits—SDHA, SDHB, SDHC, and SDHD—SDH is widely involved in metabolic regulation. Its loss of function or downregulation is a hallmark of several RCC subtypes, especially in ccRCC and SDH-deficient RCC (Aggarwal et al., 2021).

In ccRCC, metabolomic and transcriptomic analyses have revealed that SDHB expression is significantly downregulated in over 80% of tumor tissues, impairing the conversion of succinate to fumarate and leading to the depletion of downstream metabolites such as fumarate and malate. This disruption results in TCA cycle blockade and metabolite accumulation—particularly the buildup of succinate as an oncometabolite—which promotes abnormal stabilization of HIFs, a CpG island methylator phenotype (CIMP), and pro-metastatic epithelial–mesenchymal transition (EMT), collectively driving tumor progression (Sciacovelli et al., 2016). These findings suggest that SDH may serve as a valuable biomarker for early diagnosis and prognostic evaluation.

Moreover, SDH-deficient RCC has been recognized by the World Health Organization (WHO) as a distinct histological subtype, heavily reliant on immunohistochemical detection of SDHB. These tumors typically affect younger individuals and are histologically characterized by eosinophilic cytoplasm, cytoplasmic vacuoles, or inclusions. All confirmed cases show a complete loss of SDHB expression, while SDHA is usually retained (Tsai and Lee, 2019; Fuchs et al., 2022). Germline mutations—most commonly in *SDHB*—are the principal pathogenic mechanism, often resulting in multifocal, aggressive tumors with a high risk of metastasis and mortality (Fuchs et al., 2022). Therefore, loss of SDHB immunostaining serves not only as the diagnostic “gold standard” but also as a defining biomarker for SDH-deficient RCC (Table 1).

##### 3.1.2.2 FH (fumarate hydratase)

Fumarate hydratase (FH), a key enzyme in the TCA cycle, catalyzes the conversion of fumarate to malate. FH deficiency has been established as a driver event in multiple RCC subtypes, particularly in fumarate hydratase-deficient RCC associated with hereditary leiomyomatosis and renal cell carcinoma (HLRCC) syndrome (Merino et al., 2007; Yang et al., 2012). In RCC, FH downregulation or mutation induces metabolic dysregulation and leads to the accumulation of fumarate, a prototypical oncometabolite. This accumulation fosters a state of “pseudohypoxia” and epigenetic reprogramming, ultimately promoting tumorigenesis and disease progression (Isaacs et al., 2005; Selak et al., 2005).

FH-deficient RCC is characterized by massive fumarate accumulation, which inhibits α-ketoglutarate–dependent enzymes such as PHDs, TETs, and KDMs. This results in sustained HIF activation and the establishment of a CIMP phenotype, promoting angiogenesis and EMT, thereby underscoring FH’s central role in determining RCC cell fate (Sciacovelli et al., 2016). These metabolic changes also create a unique vulnerability, making FH-deficient RCC highly sensitive to therapies such as PARP inhibitors and purine metabolism inhibitors—highlighting novel opportunities for targeted treatment (Wilde et al., 2023).

Recent integrative omics studies have classified FH-deficient RCC into three distinct molecular subtypes: C1 (immune/angiogenic), C2 (WNT/Notch-activated), and C3 (proliferative/stem-like). Among these, the C1 subtype responds best to combined anti-angiogenic and immunotherapy, offering a theoretical foundation for personalized treatment strategies (Zhang et al., 2025; Chen et al., 2024). In addition, metabolomic profiling via liquid biopsy has identified two highly specific plasma biomarkers—succinyadenosine and S-(2-succinyl)cysteine—which reliably reflect FH mutation status and tumor burden, holding promise for early screening and recurrence monitoring (Zheng et al., 2023; Table 1).

##### 3.1.2.3 IDH (isocitrate dehydrogenase)

Isocitrate dehydrogenase (IDH) is a critical enzyme in the TCA cycle that catalyzes the conversion of isocitrate to α-KG, playing a central role in maintaining metabolic homeostasis. In RCC, dysregulation of IDH—particularly IDH1/2 mutation or downregulation—has been implicated in metabolic reprogramming and is increasingly recognized as a potential tumor biomarker (Shim et al., 2014).

Altered IDH activity profoundly affects RCC metabolic remodeling. Under HIF-2α and MYC activation, reverse flux through IDH mediates the reductive carboxylation of glutamine-derived α-KG to citrate, providing acetyl-CoA for lipid biosynthesis—an essential metabolic adaptation supporting rapid tumor growth (Metallo et al., 2011; Wise et al., 2008). This process is also prominently observed in FH-deficient RCC cell lines such as UOK262, further highlighting IDH’s critical role in glutamine-driven metabolic reprogramming (Mullen et al., 2011).

Ongoing studies are exploring the therapeutic potential of targeting IDH mutations in RCC. Although the overall mutation frequency of IDH in RCC is lower than in other tumor types, IDH-induced metabolites may serve as valuable biomarkers for early diagnosis and treatment monitoring (Lee et al., 2024; Yan et al., 2009; Stein et al., 2017; Table 1).

#### 3.1.3 OXPHOS

##### 3.1.3.1 PGC-1α (peroxisome proliferator-activated receptor gamma coactivator 1-α)

Peroxisome proliferator-activated receptor gamma coactivator 1-alpha (PGC-1α) is a key transcriptional coactivator that regulates mitochondrial biogenesis and oxidative phosphorylation (OXPHOS). It promotes an increase in the number of functional mitochondria and enhances the expression of OXPHOS-related proteins, thereby facilitating ATP production and elevating oxygen consumption rate (OCR) (LeBleu et al., 2014). In ccRCC, PGC-1α expression is markedly suppressed, leading to impaired mitochondrial function and a pronounced glycolytic metabolic phenotype, with significantly reduced mitochondrial pyruvate utilization by tumor cells (LaGory et al., 2015; Koh et al., 2018). Analysis of clinical specimens has demonstrated that low PGC-1α expression in ccRCC tissues is strongly associated with disease progression, metastasis, and poor patient prognosis. These findings suggest that PGC-1α may serve as a potential prognostic biomarker for patients with ccRCC (LaGory et al., 2015; Table 1).

##### 3.1.3.2 ATP synthase subunits and assembly factor

ATP synthase is a key mitochondrial enzyme complex responsible for catalyzing ATP production during OXPHOS. It is composed of multiple subunits, including structural components such as ATP5A1, ATP5G1, ATP5G2, and ATP5G3, as well as ATPAF1, an essential assembly factor (Zhou et al., 2025). In ccRCC, the expression of these ATP synthase subunits and their assembly factor is significantly downregulated, leading to impaired mitochondrial respiratory function, reduced ATP production capacity, and a compensatory increase in glycolytic metabolism (Brüggemann et al., 2017).

ATP5A1, a critical component of the F1 catalytic domain of ATP synthase, shows markedly decreased expression in ccRCC and is negatively correlated with tumor stage and histological grade, while positively associated with favorable patient prognosis (Zhou et al., 2025). Functional studies have demonstrated that silencing ATP5A1 significantly enhances the malignant phenotype of ccRCC cells, including increased proliferation, migration, and invasion, whereas ATP5A1 overexpression exerts tumor-suppressive effects. Notably, ATP5A1 may exert its anti-tumor role, at least in part, by negatively regulating the Wnt/β-catenin signaling pathway (Zhou et al., 2025).

Other ATP synthase subunits (ATP5G1, ATP5G2, ATP5G3) and the assembly factor ATPAF1 are also significantly downregulated in ccRCC, and their low expression is closely associated with poor clinical outcomes (Brüggemann et al., 2017). This downregulation is accompanied by a substantial reduction in oxygen consumption rates (OCR) of mitochondrial complexes I, II, and IV, indicating widespread impairment of OXPHOS in ccRCC cells (Brüggemann et al., 2017), which correlates with reduced OS in ccRCC patients (Brüggemann et al., 2017; Table 1).

##### 3.1.3.3 NDUFS1 (NADH dehydrogenase [ubiquinone] Fe-S protein 1)

The electron transport chain (ETC) is a core component of mitochondrial OXPHOS, and NDUFS1, a crucial subunit of complex I, plays an essential role in its function. In ccRCC, ETC-related genes, including NADH Dehydrogenase [Ubiquinone] Fe-S Protein 1 (NDUFS1), are broadly downregulated, accompanied by a marked reduction in oxygen consumption rates (OCR) of mitochondrial complexes I, II, and IV, indicating widespread mitochondrial respiratory dysfunction in renal cancer (Bezwada et al., 2023). Notably, loss of NDUFS1 expression is significantly associated with reduced overall survival in ccRCC patients (Ellinger et al., 2017; Table 1).

##### 3.1.3.4 NDUFA4L2 (NADH dehydrogenase [ubiquinone] 1 alpha subcomplex 4-like 2)

NDUFA4L2 (NADH dehydrogenase [ubiquinone] 1 alpha subcomplex 4-like 2) is a regulatory factor of mitochondrial complex I, typically induced by HIF-1α under hypoxic conditions. It suppresses oxidative phosphorylation and reduces mitochondrial ROS production, thereby promoting cellular adaptation to hypoxic microenvironments and contributing to metabolic reprogramming and therapy resistance in tumors. In contrast, NADH Dehydrogenase [Ubiquinone] 1 Alpha Subcomplex 4-Like 2 (NDUFA4L2) is significantly overexpressed in ccRCC, with more than 90% of cases exhibiting marked upregulation (Minton et al., 2016; Kubala et al., 2023). As a direct target gene of HIF-1α, NDUFA4L2 silencing enhances mitochondrial oxidative metabolism, induces excessive production of ROS, and markedly inhibits the proliferation and survival of renal cancer cells (Lucarelli et al., 2018).

Multiple clinical studies have consistently shown that high NDUFA4L2 expression is closely associated with advanced tumor stage, increased aggressiveness, and poor prognosis in ccRCC patients (Minton et al., 2016; Liu et al., 2016; Wang et al., 2017). In a histological study involving 86 ccRCC patients, the expression rate of NDUFA4L2 in tumor tissues reached 81.4%, significantly higher than in normal renal tissues (Liu et al., 2016). Its expression was strongly correlated with clinicopathological parameters and was identified as an independent adverse prognostic factor in ccRCC (Liu et al., 2016; Wang et al., 2017).

In terms of metabolic co-regulation, serine hydroxymethyltransferase 2 (SHMT2) has been shown to upregulate NDUFA4L2 expression significantly. The co-overexpression of SHMT2 and NDUFA4L2 predicts poor patient outcomes, suggesting that the SHMT2–NDUFA4L2 axis may represent a critical pathway for metabolic targeting in ccRCC (Wang et al., 2020a). Moreover, inhibition of NDUFA4L2 expression has been found to reduce neutral lipid accumulation in renal tissues and suppress the expression of ccRCC biomarkers such as CA9 and ENO, thereby impeding tumor progression (Laursen et al., 2021; Table 1).

##### 3.1.3.5 UQCRC1 (ubiquinol-cytochrome c reductase core protein 1) and UQCRH (ubiquinol–cytochrome c reductase hinge protein)

Dysfunction of complex III in the mitochondrial electron transport chain (ETC) plays a critical role in the pathogenesis of ccRCC and exhibits molecular features that may be valuable for diagnosis and subtyping. UQCRC1 (ubiquinol-cytochrome c reductase core protein 1) is a core subunit of mitochondrial respiratory complex III, essential for electron transfer from ubiquinol to cytochrome c and for maintaining efficient oxidative phosphorylation and ATP production. UQCRC1 is consistently downregulated in ccRCC and is significantly associated with patient survival (Ellinger et al., 2016; Coffey and Simon, 2024). Systematic analyses have demonstrated persistent low expression of UQCRC1 across multiple independent ccRCC microarray cohorts, with downregulation confirmed at both the protein and mRNA levels (Ellinger et al., 2016). TCGA data further reveal that decreased UQCRC1 expression is frequently accompanied by hypermethylation in the promoter region, suggesting that its silencing may be epigenetically regulated (Ellinger et al., 2016).

UQCRH (ubiquinol–cytochrome c reductase hinge protein) is a hinge protein component of mitochondrial ETC complex III, involved in electron transfer between cytochrome c1 and cytochrome c. Multiple studies have shown that UQCRH undergoes promoter hypermethylation, mRNA downregulation, and functional inactivation—changes that are positively associated with increased tumor aggressiveness (Luo et al., 2020; Miyakuni et al., 2022). Immunohistochemical analysis revealed that UQCRH expression is significantly lower in ccRCC tissues compared to adjacent non-tumor tissues. Its silencing impairs mitochondria-mediated cytochrome c release and promotes primary tumor formation (Miyakuni et al., 2022). In the KMRC2 renal cancer cell line, ectopic overexpression of UQCRH significantly restores mitochondrial membrane potential, enhances oxygen consumption rate, suppresses the Warburg effect, inhibits cell proliferation, and promotes apoptosis (Luo et al., 2020; Table 1).

#### 3.1.4 Pentose phosphate pathway

##### 3.1.4.1 G6PD (glucose-6-phosphate dehydrogenase)

Glucose-6-phosphate dehydrogenase (G6PD) is the key rate-limiting enzyme of the pentose phosphate pathway (PPP), catalyzing the conversion of glucose-6-phosphate into ribose-5-phosphate and nicotinamide adenine dinucleotide phosphate (NADPH). This reaction is essential for nucleotide biosynthesis and the maintenance of cellular redox homeostasis (Wettersten, 2020). Data from The Cancer Genome Atlas (TCGA) indicate that G6PD expression is significantly upregulated in both ccRCC and papillary RCC (pRCC), and this overexpression is strongly associated with increased tumor aggressiveness and poor patient prognosis (Zhang et al., 2022; Liu et al., 2023).

Furthermore, extensive clinical evidence supports the prognostic value of G6PD in RCC. Retrospective analyses have demonstrated that high G6PD expression in renal tumor tissues serves as an independent risk factor for poor postoperative survival outcomes (Zhang et al., 2022). A pan-cancer analysis further revealed that elevated G6PD expression is not only linked to worse prognosis in RCC but also shows a significant positive correlation with immune cell infiltration across various cancer types, suggesting a potential role for G6PD in modulating the tumor immune microenvironment (Liu et al., 2023; Table 1).

##### 3.1.4.2 G6PI (glucose-6-phosphate isomerase)

Glucose-6-phosphate isomerase (G6PI), also known as phosphoglucose isomerase (GPI), was originally identified as a key enzyme in the glycolytic pathway, catalyzing the interconversion of glucose-6-phosphate to fructose-6-phosphate (Lucarelli et al., 2015b). Elevated GPI expression is significantly associated with poor prognosis in patients with ccRCC. In a study involving 180 ccRCC tissue specimens, tissue microarray immunohistochemistry (TMA-IHC) and immunofluorescence (IF) analyses revealed that GPI and autocrine motility factor receptor (AMFR) signaling were both markedly enhanced in tumor tissues compared to adjacent non-tumor tissues. Moreover, GPI and AMFR were co-localized on the plasma membrane, suggesting that the GPI/AMF signaling axis may play a critical role in ccRCC progression (Lucarelli et al., 2015b). Kaplan–Meier survival analysis demonstrated that patients with high GPI expression had significantly worse cancer-specific survival (CSS) and progression-free survival (PFS) compared to those with low GPI expression. Specifically, the 5-year CSS rate was only 58.8% in the high GPI expression group, compared to 92.1% in the low expression group. Similarly, the 5-year PFS was 56.8% in the high-expression cohort, markedly lower than 93.3% in the low-expression cohort (Lucarelli et al., 2015b). Multivariate analysis further confirmed that high GPI expression is an independent adverse prognostic factor for both CSS and PFS in ccRCC, highlighting its potential utility as a prognostic biomarker for assessing tumor aggressiveness and risk of disease progression (Lucarelli et al., 2015b; Table 1).

##### 3.1.4.3 TKT (transketolase)

Transketolase (TKT) is a key enzyme in the non-oxidative branch of the PPP, catalyzing the reversible conversion between pentose sugars and glycolytic intermediates. This reaction supplies essential metabolites for nucleotide biosynthesis and supports the rapid proliferation of tumor cells (Langbein et al., 2008). In ccRCC, both the oxidative and non-oxidative branches of the PPP are significantly upregulated as cancer cells adapt to metabolic and oxidative stress, with TKT activity serving as a hallmark of non-oxidative PPP activation (Lucarelli et al., 2015a).

Studies using The Cancer Genome Atlas (TCGA) have revealed a global upregulation of the PPP, including high TKT expression, which is strongly associated with poor prognosis in patients with aggressive ccRCC (Ricketts et al., 2018). Additional research has shown that TKT expression is induced by miR-146a-5p and is higher in advanced RCC tumors compared to early-stage lesions. Elevated TKT levels are correlated with shortened patient survival, underscoring its prognostic significance (Bogusławska et al., 2019). Specifically, PPP-related metabolites such as ribose-5-phosphate and xylulose-5-phosphate are significantly elevated in renal tumor tissues, indicating enhanced TKT activity that provides a sufficient supply of metabolic intermediates to meet the demands of nucleotide biosynthesis and sustain tumor growth and proliferation (Langbein et al., 2008).

Furthermore, a study by Langbein et al. demonstrated that TKT activity is significantly higher in metastatic compared to primary RCC tissues. This elevation is particularly pronounced in highly invasive and lethal tumors, where non-oxidative glucose fermentation is markedly increased. These findings suggest that TKT-mediated activation of the non-oxidative PPP may represent a key metabolic event driving RCC invasion and metastasis (Langbein et al., 2008; Table 1).

#### 3.1.5 Gluconeogenesis

##### 3.1.5.1 PCK1 (phosphoenolpyruvate carboxykinase 1)

Phosphoenolpyruvate carboxykinase 1 (PCK1) is one of the key rate-limiting enzymes in the gluconeogenic pathway, primarily functioning by catalyzing the conversion of oxaloacetate to phosphoenolpyruvate. In ccRCC, PCK1 expression is significantly negatively correlated with tumor progression, lactate dehydrogenase A (LDHA) expression in primary ccRCC tissues, and uptake of 18F-FDG, suggesting that PCK1 suppresses glycolytic activity by downregulating LDHA expression (Shi et al., 2020). Further studies have revealed that PCK1 reduces LDHA protein stability through post-translational modifications, thereby significantly inhibiting aerobic glycolysis in ccRCC cells. This metabolic suppression impedes tumor cell proliferation, metastasis, and tumor formation in xenograft models (Shi et al., 2020; Table 1).

##### 3.1.5.2 G6PC (glucose-6-phosphatase)

Glucose-6-phosphatase (G6PC), which catalyzes the conversion of glucose-6-phosphate into free glucose for release into the bloodstream, is significantly downregulated in ccRCC tissues. This reduction is strongly associated with decreased overall survival in patients (Xu et al., 2020c). A multi-cohort clinical study involving 718 patients demonstrated that low G6PC expression is a significant predictor of poorer survival outcomes and increased tumor aggressiveness (Xu et al., 2020c; Table 1).

##### 3.1.5.3 FBP1 (fructose-1,6-bisphosphatase 1)

Fructose-1,6-bisphosphatase 1 (FBP1), which catalyzes the conversion of fructose-1,6-bisphosphate to fructose-6-phosphate, functions as an antagonist to glycolysis (Moore et al., 2012). In ccRCC, FBP1 expression is markedly reduced, with its loss observed in nearly all RCC tumor tissues (Li et al., 2014). Studies have shown that depletion of FBP1 strongly promotes the Warburg effect, thereby enhancing the proliferation and invasiveness of renal cancer cells (Li et al., 2014; Liao et al., 2020). Furthermore, low FBP1 expression is significantly associated with poor prognosis, while ectopic expression of FBP1 has been shown to effectively suppress tumor growth in xenograft models (Ju et al., 2022; Table 1).

### 3.2 Biomarkers in lipid metabolism

#### 3.2.1 Lipogenesis

##### 3.2.1.1 FASN (fatty acid synthase)

Fatty acid synthase (FASN) is a multifunctional enzyme that catalyzes the synthesis of long-chain fatty acids from acetyl-CoA and malonyl-CoA. It serves as the rate-limiting enzyme in the *de novo* lipogenesis (DNL) pathway and relies on acetyl-CoA and NADPH, which are primarily provided by glucose metabolism and the PPP in tumor cells.

In ccRCC, FASN is widely overexpressed and has emerged as a central event in tumor metabolic reprogramming. Early studies revealed significant accumulation of cholesterol esters and long-chain fatty acids in ccRCC tumor tissues, accompanied by upregulation of lipogenic genes such as FASN and SCD1 (Gebhard et al., 1987). Metabolomic profiling has further demonstrated elevated levels of lipid intermediates such as acylcarnitines in ccRCC, which correlates with the downregulation of FAO enzymes, suggesting a metabolic shift favoring lipid storage over utilization (Ganti et al., 2012b).

Large-scale clinical analyses have shown that high FASN expression is closely associated with advanced tumor stage, increased invasiveness, and poor prognosis (Xu et al., 2020b). In both TCGA and FUSCC cohorts, elevated FASN expression significantly correlated with advanced AJCC stage, T stage, and N stage, and was negatively associated with PFS and OS (Xu et al., 2020b). Further studies also revealed a positive correlation between FASN expression and visceral adipose tissue volume (VAT%), suggesting that host metabolic status may influence tumor behavior (Xu et al., 2020b). Emerging evidence suggests that perirenal adipose tissue may engage in paracrine cross-talk with ccRCC via adipokines (e.g., leptin, adiponectin, chemerin, apelin) (Grigoraş and Amalinei, 2025), pro-inflammatory cytokines such as IL-6 (Wang et al., 2022a), and direct lipid transfer (Song et al., 2024), while tumor cells can reprogram adjacent adipocytes into cancer-associated adipocytes that secrete bioactive factors to sustain lipogenesis (Bruna et al., 2019); these interactions may even be reflected in imaging markers of peritumoral inflammation, such as the CT fat attenuation index (Yang, 2022; Table 2).

**TABLE 2:** Functions and clinical biomarker value of molecules involved in lipid metabolism reprogramming in RCC.

Molecule	Function	Biomarker role or clinical value	References
FASN	Rate-limiting enzyme of *de novo* fatty acid synthesis, generates long-chain fatty acids.	Overexpression is associated with advanced stage, tumor aggressiveness, and poor prognosis.	Xu et al. (2020b)
ACLY	Converts citrate to acetyl-CoA, providing substrate for lipid biosynthesis.	High expression correlates with decreased OS and PFS, serving as a negative prognostic biomarker.	Yin et al. (2024)
ACC	Rate-limiting enzyme of *de novo* lipogenesis, catalyzes acetyl-CoA to malonyl-CoA.	Overexpression is associated with AMPK dysregulation, lipid accumulation, and tumor progression; functions in metabolic regulation and prognostic evaluation.	Tan et al. (2023)
SCD	Catalyzes the formation of MUFAs from SFAs, supporting lipid droplet formation and membrane fluidity.	SCD1 overexpression promotes cell survival and lipid droplet formation, correlating with poor prognosis. SCD5 downregulation may exert tumor-suppressive effects. SCD1 is a potential imaging and therapeutic response biomarker.	Melana et al. (2021), Ganner et al. (2023), Li et al. (2023a)
FADS	Catalyzes synthesis of PUFAs such as AA, EPA, and DHA.	FADS1 overexpression leads to PUFA-phospholipid accumulation, altering membrane structure and signaling pathways.	Saito et al. (2016)
CPT1A	Rate-limiting enzyme for fatty acid β-oxidation, facilitating mitochondrial entry.	Downregulation leads to lipid droplet accumulation and metabolic reprogramming. Restored expression suppresses tumor growth, making it a prognostic indicator and therapeutic target.	Du et al. (2017)
PLIN2	Lipid droplet-associated protein that regulates droplet stability and ER homeostasis.	High expression correlates with advanced stage and grade; downregulation enhances ccRCC cell proliferation, invasion, and migration.	Cao et al. (2018)
ECHS1	β-oxidation enzyme regulating short-chain fatty acid metabolism.	Differentiates early-stage tumors from adjacent tissue; persistently downregulated in cancer with diagnostic sensitivity. Upregulation inhibits mTOR pathway, offering therapeutic and stratification potential.	Wang et al. (2020b), Hu et al. (2022)
FABP	Fatty acid transport proteins involved in intracellular lipid homeostasis.	FABP5 promotes EMT and AKT pathway activation, indicating poor prognosis. Elevated serum/urinary FABP4 serves as a non-invasive diagnostic biomarker.	Lv et al. (2019), Wu et al. (2019), Wu et al. (2020), La Civita et al. (2025)
CD36	Mediates uptake of exogenous fatty acids.	High expression correlates with advanced TNM stage, increased VAT%, and shorter PFS/OS.	Xu et al. (2019b)
SCARB1	Mediates HDL-cholesterol uptake.	Overexpression activates the PI3K/AKT pathway, promoting proliferation and survival.	Riscal et al. (2021)
DHA	An omega-3 PUFA with anti-tumor properties.	Low serum DHA levels reflect metastatic risk and poor cancer-specific survival. Has synergistic effects with targeted therapy and may assist in treatment monitoring.	Tasaki et al. (2016)

##### 3.2.1.2 ACLY (ATP citrate lyase)

ATP citrate lyase (ACLY) is a critical metabolic enzyme that links glycolysis to lipid biosynthesis by catalyzing the conversion of cytosolic citrate, derived from the mitochondria, into acetyl-CoA and oxaloacetate. Acetyl-CoA is a central substrate in the DNL pathway, supporting the biosynthesis of lipids, sterols, and acylated proteins (Teng et al., 2018). Recent studies have reported that ACLY is highly expressed in ccRCC and is strongly associated with poor clinical outcomes. Analyses from GEPIA2, GEO, and UALCAN databases revealed significant upregulation of ACLY mRNA in ccRCC tissues, which was further validated at the protein level by immunohistochemistry (Yin et al., 2024). Kaplan–Meier survival analyses demonstrated that high ACLY expression is significantly associated with reduced OS and PFS, supporting its potential as a prognostic biomarker for poor outcomes in ccRCC (Yin et al., 2024; Table 2).

##### 3.2.1.3 ACC (Acetyl-CoA carboxylase)

Acetyl-CoA carboxylase (ACC) is the rate-limiting enzyme in the DNL pathway, catalyzing the carboxylation of acetyl-CoA to produce malonyl-CoA (2013). Due to the high lipid synthesis demand of rapidly proliferating tumor cells, ACC expression is frequently upregulated. Gene expression analyses have shown significant elevation of ACC mRNA levels in ccRCC, which is closely associated with unfavorable prognosis and a marked decrease in OS (Tan et al., 2023).

At the metabolic regulation level, the balance between ACC and AMP-activated protein kinase (AMPK) is crucial for coordinating lipid synthesis and oxidation. As an energy sensor, AMPK directly inhibits ACC to suppress lipogenesis (Tan et al., 2023). However, multi-omics studies have shown that in ccRCC, ACC is upregulated while AMPK activity is downregulated, suggesting that this imbalance facilitates lipid accumulation and tumor growth (Tan et al., 2023).

Furthermore, ACC is also a key downstream target of the AMPK signaling axis. In diabetic nephropathy models, AMPK activation inhibits ACC phosphorylation and reduces lipid deposition. Empagliflozin has been shown to delay lipid-induced renal damage by activating the AdipoR1/AMPK/p-ACC pathway (Zhang et al., 2021b; Table 2).

##### 3.2.1.4 SCD (Stearoyl-CoA desaturase)

Stearoyl-CoA desaturase (SCD) is the rate-limiting enzyme responsible for converting saturated fatty acids (SFAs) into monounsaturated fatty acids (MUFAs), primarily producing oleic acid (C18:1 n-9), a critical component of membrane phospholipids, triglycerides, and cholesteryl esters (Enoch et al., 1976). In ccRCC, SCD1 upregulation is considered a key factor in maintaining this distinct lipid metabolic phenotype (Lucarelli et al., 2020).

Studies have shown that SCD1 is consistently overexpressed in ccRCC cell lines and primary tumors, with its expression negatively correlated with patient survival (Wang et al., 2016a; von Roemeling et al., 2013). Elevated SCD1 promotes oleic acid synthesis, enhances lipid droplet formation, stabilizes the endoplasmic reticulum, and supports tumor cell proliferation and survival (Melana et al., 2021).

In addition to SCD1, SCD5 has also been implicated in ccRCC. Interestingly, under conditions of VHL deficiency or hypoxia, SCD5 is significantly downregulated in ccRCC tissues and model organisms. Restoration of SCD5 expression inhibits tumor cell proliferation, suggesting a potential tumor-suppressive role that contrasts with the pro-oncogenic effects of SCD1 (Ganner et al., 2023).

Furthermore, a novel PET radiotracer [^11^C]SSI-4, has been developed to visualize SCD1 expression *in vivo*. This tracer shows specific accumulation in SCD1-high tissues and RCC xenografts, offering promising potential for diagnostic imaging and therapeutic response assessment (Li et al., 2023a), highlighting the broad clinical relevance of SCD in RCC management (Table 2).

##### 3.2.1.5 FADS (The fatty acid desaturase)

The fatty acid desaturase (FADS) family, including FADS1 and FADS2, catalyzes critical steps in the biosynthesis of polyunsaturated fatty acids (PUFAs) from dietary precursors. These enzymes are involved in the conversion of linoleic acid and α-linolenic acid into arachidonic acid (AA), eicosapentaenoic acid (EPA), and docosahexaenoic acid (DHA) (Chilton et al., 2017). Beyond structural roles in membranes, PUFAs regulate signaling, inflammation, and cell death through bioactive lipid mediators. Notably, AA-derived PGE2 has been linked to cancer progression, while EPA and DHA metabolites demonstrate anti-tumor properties (Heravi et al., 2022).

FADS1 is commonly overexpressed in ccRCC, leading to increased PUFA levels and the accumulation of PUFA-containing phospholipids (e.g., phosphatidylethanolamine [PE], phosphatidylcholine [PC]) within tumor tissues, especially in high-grade tumors (Saito et al., 2016). Lipidomic studies further reveal that despite FADS1 overexpression, free PUFAs are reduced in tumor tissues, suggesting their incorporation into membrane lipids, potentially affecting membrane fluidity and signal transduction (Zou et al., 2019; Table 2).

#### 3.2.2 Lipolysis and β-oxidation

##### 3.2.2.1 CPT1A (carnitine palmitoyltransferase 1A)

Carnitine palmitoyltransferase 1A (CPT1A) is the rate-limiting enzyme in FAO, regulating the mitochondrial import of long-chain fatty acids and playing a key role in tumor cell energy metabolism and growth (Melone et al., 2018). Under physiological conditions, CPT1A-mediated mitochondrial FA transport generates ATP and supports tumor cell adaptation to metabolic stress. However, in contrast to other malignancies such as lung, prostate, and breast cancers, where CPT1A is frequently upregulated, CPT1A expression is markedly downregulated in ccRCC (Du et al., 2017).

Further studies revealed that CPT1A repression in ccRCC is driven by HIF activation due to VHL loss. HIF directly targets and inhibits CPT1A transcription, preventing mitochondrial FAO and leading to cytoplasmic lipid droplet accumulation—a hallmark of ccRCC pathology (Du et al., 2017). Restoration of CPT1A expression in ccRCC cells significantly reduces lipid accumulation and suppresses tumor growth *in vivo*, indicating that CPT1A downregulation contributes to poor prognosis (Du et al., 2017). Recent studies further demonstrated that CPT1A suppresses cholesterol uptake and lipid accumulation through the PPARα/CD36 axis and inhibits Akt phosphorylation, thereby restraining tumor progression, providing additional support for CPT1A as a potential therapeutic target in ccRCC (Yang et al., 2022; Table 2).

##### 3.2.2.2 PLIN2 (perilipin 2)

Perilipin 2 (PLIN2), a key lipid droplet-associated protein, is significantly upregulated in ccRCC and is transcriptionally regulated by HIF-2α. Specifically, HIF-2α promotes PLIN2 expression, facilitating lipid droplet accumulation near the endoplasmic reticulum (ER), which in turn helps maintain ER homeostasis and enhances cellular tolerance to cytotoxic stress in ccRCC cells (Qiu et al., 2015).

Clinical data analyses have shown that PLIN2 is highly expressed in ccRCC tissues and is positively correlated with multiple clinicopathological parameters, including tumor stage and grade. Notably, PLIN2 expression is inversely correlated with patient survival; high PLIN2 levels have been identified as an independent favorable prognostic marker. Conversely, PLIN2 downregulation enhances ccRCC cell proliferation, invasion, and migration, underscoring its dual role in both tumor biology and clinical prognosis (Cao et al., 2018; Table 2).

##### 3.2.2.3 ECHS1 (Enoyl-CoA hydratase short chain 1)

Enoyl-CoA hydratase short chain 1 (ECHS1) is a key mitochondrial enzyme in fatty acid β-oxidation, catalyzing the hydration of short-chain enoyl-CoA and playing a vital role in maintaining lipid metabolic homeostasis. In ccRCC, ECHS1 expression is significantly downregulated, suggesting its involvement as a regulatory factor in tumor initiation and progression (Hu et al., 2022). Proteomic analyses have shown that ECHS1 remains consistently underexpressed across different tumor stages and effectively distinguishes early-stage tumors from adjacent normal tissues, with an area under the curve (AUC) exceeding 0.7—demonstrating high diagnostic sensitivity and specificity. Functional studies further reveal that upregulation of ECHS1 suppresses tumor cell proliferation and migration via inhibition of the mTOR signaling pathway, underscoring its potential as a therapeutic target in ccRCC (Wang et al., 2020b; Table 2).

#### 3.2.3 Lipid transport

##### 3.2.3.1 FABP (fatty acid-binding proteins)

Fatty acid-binding proteins (FABPs) are a family of low-molecular-weight (14–15 kDa) intracellular lipid chaperones that mediate the transport and storage of free fatty acids (FFAs) (Koundouros and Poulogiannis, 2020). Given the elevated lipid demands of cancer cells, FABPs are aberrantly expressed in various malignancies including pancreatic, colorectal, and ovarian cancers, where they contribute to tumor progression (Kawaguchi et al., 2016; Senga et al., 2018; Gharpure et al., 2018).

In ccRCC, FABP5 is significantly upregulated and strongly associated with tumor progression, metastasis, and poor clinical outcomes (Lv et al., 2019; Wu et al., 2019; Wu et al., 2020). *In vitro* and *in vivo* experiments confirm that FABP5 knockdown significantly reduces ccRCC cell proliferation and clonogenic potential while inhibiting epithelial–mesenchymal transition (EMT), indicating its oncogenic role in ccRCC biology (Wu et al., 2019). Mechanistically, FABP5 appears to exert its tumor-promoting effects through activation of the PI3K/AKT signaling pathway; inhibition of this pathway attenuates FABP5-driven proliferation (Lv et al., 2019).

Additionally, serum FABP4 levels are markedly elevated in ccRCC patients, suggesting its potential as a non-invasive diagnostic biomarker (La Civita et al., 2025). *In vitro* studies further show that adipocyte-derived FABP4 enhances ccRCC cell migration via partial activation of the ERK signaling pathway. Notably, this effect can be reversed by the FABP4-specific inhibitor BMS309403 (La Civita et al., 2025). Proteomic analysis of patient urine samples also revealed significantly elevated urinary FABP4 levels in ccRCC, supporting its potential utility as a non-invasive biomarker for early detection and disease monitoring (Yang et al., 2023; Table 2).

##### 3.2.3.2 CD36

CD36, a multifunctional transmembrane glycoprotein and fatty acid translocase, facilitates the uptake of exogenous fatty acids (FAs), thereby supporting the lipid demands of rapidly proliferating tumor cells. It has been widely reported as a prognostic biomarker in various cancers (Enciu et al., 2018). In ccRCC, CD36 is significantly upregulated in both tumor tissues and cell lines.

CD36 mRNA expression is markedly higher in ccRCC tissues compared to adjacent normal tissues, and its overexpression is strongly associated with advanced TNM stage, increased visceral adipose tissue percentage (VAT%), and poorer PFS and OS. These findings suggest that CD36 overexpression is not only an independent predictor of poor prognosis in ccRCC patients but may also reflect the host’s abdominal fat distribution (Xu et al., 2019b; Table 2).

##### 3.2.3.3 SCARB1 (scavenger receptor class B type 1)

SCARB1 (scavenger receptor class B type 1) is a HDL receptor that mediates the selective uptake of cholesteryl esters and plays a critical role in cholesterol homeostasis, lipid metabolism, and steroidogenesis. Genes involved in endogenous cholesterol biosynthesis are significantly downregulated in ccRCC, indicating a heavy reliance on exogenous cholesterol uptake for tumor growth (Riscal et al., 2021). Further investigations confirmed that inhibiting SCARB1-mediated HDL cholesterol uptake significantly suppresses ccRCC cell proliferation and survival, induces cell cycle arrest and apoptosis, and is accompanied by elevated intracellular ROS levels and marked attenuation of the PI3K/AKT signaling pathway (Riscal et al., 2021; Coffey and Simon, 2024). These findings identify SCARB1 as a promising therapeutic target for ccRCC (Table 2).

#### 3.2.4 Metabolic derivatives

##### 3.2.4.1 DHA (docosahexaenoic acid)

Docosahexaenoic acid (DHA), a prototypical omega-3 PUFA, is primarily obtained from the diet and synthesized endogenously via a series of elongation and desaturation reactions from the essential fatty acid α-linolenic acid (ALA) (Heravi et al., 2022). Recent studies have demonstrated that serum DHA levels are significantly reduced in patients with RCC and are closely associated with metastatic status. Preoperative serum analyses revealed that patients with metastatic RCC exhibit markedly lower DHA levels compared to those without metastases. Moreover, patients with DHA levels below the median showed significantly shorter cancer-specific survival, suggesting that serum DHA may serve as a potential prognostic biomarker for poor outcomes in RCC (Tasaki et al., 2016).

Beyond its prognostic value, DHA also holds therapeutic potential. In both RCC cell lines and animal models, DHA synergistically enhances the anticancer efficacy of the multikinase inhibitor regorafenib. Regorafenib suppresses soluble epoxide hydrolase (sEH), resulting in elevated levels of DHA-derived epoxydocosapentaenoic acids (EDPs), which exhibit potent anti-angiogenic activity and significantly inhibit tumor angiogenesis and invasiveness. These findings support the potential clinical utility of DHA as an adjuvant agent in the treatment of advanced RCC (Kim et al., 2016). Furthermore, in syngeneic mouse models of RCC treated with anlotinib, serum DHA levels changed in a highly sensitive and specific manner in response to treatment, indicating its promise as a pharmacodynamic biomarker (Du et al., 2022; Table 2).

### 3.3 Biomarkers in amino acid metabolism

#### 3.3.1 Glutamine metabolism

##### 3.3.1.1 SLC1A5 (solute carrier family 1 member 5)

SLC1A5 (Solute Carrier Family 1 Member 5), also known as ASCT2, is a sodium-dependent neutral amino acid transporter primarily responsible for glutamine uptake. ccRCC cells exhibit glutamine addiction, which is mediated by SLC1A5. The imported glutamine is converted to glutamate by glutaminase (GLS), which is subsequently transformed into α-KG to replenish the tricarboxylic acid (TCA) cycle and support lipid biosynthesis, antioxidant defense, and nucleotide production (Hassanein et al., 2013). Key metabolic regulators, HIF2 and c-Myc, cooperatively induce the expression of SLC1A5 and GLS, promoting glutamine-driven fatty acid synthesis and glutathione generation, thereby enhancing proliferation and antioxidant capacity in ccRCC cells (Shroff et al., 2015).

Multiple studies have reported significantly elevated SLC1A5 expression in ccRCC tissues and cell lines, correlating with poor survival outcomes, advanced TNM stage, lymph node metastasis, and higher Fuhrman grade. SLC1A5 has been identified as an independent adverse prognostic factor for both OS and RFS (Liu et al., 2015; Kawakami et al., 2022; Qin et al., 2024). Metabolic reprogramming analyses further suggest that SLC1A5 overexpression is strongly associated with sunitinib resistance, implicating its role in targeted therapy resistance in RCC (Sato et al., 2020; Table 3).

**TABLE 3:** Functions and clinical biomarker value of molecules involved in amino acid metabolism reprogramming in RCC.

Molecule	Function	Biomarker role or clinical value	References
SLC1A5	Sodium-dependent neutral amino acid transporter, mediates glutamine uptake.	High expression correlates with advanced TNM stage, lymph node metastasis, and sunitinib resistance.	Liu et al. (2015), Kawakami et al. (2022), Qin et al. (2024)
GLS	Converts glutamine to glutamate, supporting TCA cycle and lipid/nucleotide synthesis.	Overexpressed in ccRCC and FH-deficient RCC, promoting metabolic adaptation and tumor growth.	Emberley et al. (2021)
GPX	GSH-dependent oxidoreductases; GPX4 regulates lipid peroxidation and ferroptosis.	GPX1 overexpression is associated with high-grade, metastatic, and poor survival tumors. GPX4 inhibition induces ferroptosis and may serve as a diagnostic, prognostic, and therapeutic biomarker.	Cheng et al. (2019), Chen et al. (2020), Ren et al. (2023)
IDO1	Converts tryptophan to kynurenine, mediating immunosuppressive metabolism.	Expressed in endothelial cells; high expression predicts sensitivity to PD-1 therapy. Co-expressed with PD-L1 in certain RCC subtypes.	Seeber et al. (2018), Kiyozawa et al. (2020)
TDO2	Tumor-expressed kynurenine-producing enzyme that drives immune suppression.	High expression is associated with reduced CD8^+^ T cell infiltration, poor OS, and predictive value for ICI therapy response.	Sumitomo et al. (2021), Pham et al. (2021), Yao et al. (2024)
ASS1	Catalyzes the formation of argininosuccinate from citrulline and aspartate.	Loss of expression leads to arginine dependence, associated with high-grade, aggressive tumors and poor survival.	Khare et al. (2021), Wang et al. (2019)
ARG2	Mitochondrial arginase that converts arginine into ornithine and urea.	Downregulation creates a metabolic vulnerability; however, in specific contexts, re-expression can suppress tumor growth. High expression may also contribute to immune evasion via L-arginine depletion and T cell suppression.	Ochocki et al. (2018)

##### 3.3.1.2 GLS (glutaminase)

Glutaminase (GLS) catalyzes the deamination of glutamine to produce glutamate, facilitating multiple downstream metabolic processes including TCA cycle replenishment, lipid synthesis, and glutathione production (Hassanein et al., 2013). Elevated GLS activity has been positively associated with tumor growth and is widely overexpressed in ccRCC tissues (Emberley et al., 2021), making it a promising target for metabolism-based therapies. GLS expression is regulated by oncogenic factors such as HIF2α and c-Myc. Under co-activation of HIF2 and MYC, glutamine is converted via GLS to glutamate, which is further transformed by glutamate dehydrogenase (GDH) into α-KG, feeding into the TCA cycle, or reductively carboxylated by isocitrate dehydrogenase (IDH) to generate isocitrate and acetyl-CoA for lipid biosynthesis (Shroff et al., 2015). Importantly, GLS plays a central role in glutamine-dependent lipogenesis, and its activity directly impacts lipid metabolic reprogramming (Lobo et al., 2000). Moreover, GLS is also upregulated in FH-deficient RCC, where the lncRNA MIR4435-2HG promotes GLS1 expression through interaction with STAT1, establishing a key regulatory axis for metabolic adaptation in this RCC subtype (Zhu et al., 2024; Table 3).

##### 3.3.1.3 GPX (glutathione peroxidase)

The GPX (glutathione peroxidase) family comprises glutathione-dependent antioxidant enzymes that reduce hydrogen peroxide and organic hydroperoxides, thereby protecting cells from lipid peroxidation and oxidative stress. Multi-omics studies have revealed significant enrichment of both GSH and its oxidized form (GSSG) in ccRCC cells. GPX1 is notably upregulated across several studies, with its expression correlating with higher tumor grade, advanced stage, and metastatic potential, underscoring its protective role in maintaining redox balance in tumor cells (Hakimi et al., 2016; Wettersten et al., 2015). Additionally, GPX1 overexpression is significantly associated with shorter overall survival, distant metastasis, and disease progression. ROC curve analysis has confirmed its robust diagnostic utility (Cheng et al., 2019; Chen et al., 2020). Elevated GPX1 levels have also been observed across multiple RCC subtypes (KICH, KIRP, KIRC), with strong associations to immune infiltration, molecular subtypes, and clinical staging (Chen et al., 2020).

Beyond GPX1, GPX4 plays a more direct role in regulating ferroptosis. As a lipid hydroperoxide reductase, GPX4 utilizes GSH to neutralize lipid peroxides on the cell membrane. It is ubiquitously upregulated in ccRCC tissues, and its inhibition markedly increases lipid peroxide sensitivity, triggering ferroptosis and reducing cell proliferation (Zou et al., 2019). Immunohistochemical and survival analyses have confirmed that elevated GPX4 levels are closely associated with tumor size, metastatic risk, and shorter PFS, highlighting its prognostic significance (Ren et al., 2023; Table 3).

#### 3.3.2 Tryptophan metabolism

##### 3.3.2.1 IDO1 (indoleamine 2,3-dioxygenases)/TDO2 (tryptophan 2,3-dioxygenase)

Tryptophan, an essential amino acid, is predominantly metabolized (approximately 95%) via the kynurenine (Kyn) pathway, catalyzed by rate-limiting enzymes including indoleamine 2,3-dioxygenases (IDO1/2) and tryptophan 2,3-dioxygenase (TDO2) (Wettersten, 2020). These enzymes convert tryptophan into immunosuppressive metabolites such as kynurenine, which in turn suppress T cell activity, promote Treg infiltration, and impair anti-tumor immunity, contributing to poor prognosis in ccRCC (Seeber et al., 2018; Sumitomo et al., 2021).

IDO1 is widely overexpressed in ccRCC, predominantly localized in tumor vasculature rather than cancer cells, and is associated with reduced CD8^+^ T cell infiltration (Seeber et al., 2018). In mRCC patients treated with PD-1 inhibitor nivolumab, IDO1 expression (>10%) significantly predicts prolonged PFS and correlates positively with CD8^+^ T cell infiltration, whereas PD-L1 lacks such predictive value, suggesting IDO1 as a more promising immunotherapeutic biomarker (Seeber et al., 2018). Furthermore, co-expression of IDO1 and PD-L1 is notably enriched in sarcomatoid/rhabdoid RCC and is associated with dense CD3+/CD4+/CD8+ T cell infiltration and worse PFS (Kiyozawa et al., 2020).

In contrast, recent studies suggest TDO2 may play a more dominant role in ccRCC. TDO2 is primarily expressed in tumor cells rather than endothelial cells and is linked to Kyn accumulation, reduced CD8^+^ T cells, increased invasiveness, and poor OS (Sumitomo et al., 2021; Pham et al., 2021; Yao et al., 2024). TDO2 expression strongly correlates with intratumoral Kyn levels and is predictive of immune checkpoint inhibitor (ICI) treatment response, identifying it as a potential biomarker for ICI efficacy (Sumitomo et al., 2021).

Moreover, expression of tryptophan metabolism–related genes (TMRs) may enable molecular classification of ccRCC. A six-gene prognostic model derived from TCGA and ICGC data—including TDO2, KMO, and CYP1B1—demonstrated that TDO2 is markedly upregulated in high-risk subtypes, which are associated with poorer prognosis (Yao et al., 2024; Table 3).

#### 3.3.3 Arginine metabolism

##### 3.3.3.1 ASS1 (argininosuccinate synthetase 1)

Argininosuccinate synthetase 1 (ASS1) is a key rate-limiting enzyme in arginine biosynthesis, catalyzing the conversion of citrulline and aspartate into argininosuccinate, playing a vital role in the urea cycle (Wettersten et al., 2017). Multiple proteomic and transcriptomic studies consistently report that ASS1 is significantly downregulated or entirely lost in ccRCC tissues, while its expression remains high in adjacent normal renal tubular epithelium (Khare et al., 2021). Loss of ASS1 results in tumor cells becoming auxotrophic for arginine, dependent on exogenous supply—a metabolic vulnerability known as “arginine auxotrophy” (Rabinovich et al., 2015).

In addition to its metabolic role, ASS1 may serve as a prognostic biomarker. Reduced ASS1 expression is strongly associated with higher tumor grade, increased aggressiveness, and decreased survival (Khare et al., 2021; Wang et al., 2019). Co-expression of ASS1 and its partner enzyme ASL *in vitro* and *in vivo* models can restore nitric oxide synthesis and aspartate metabolism, significantly inhibiting tumor proliferation (Khare et al., 2021). Furthermore, stabilization of HIF under VHL-deficient conditions suppresses mitochondrial function and limits aspartate availability. In this context, ASS1 expression is transcriptionally repressed to minimize aspartate diversion to the urea cycle, thereby favoring pyrimidine and nucleotide biosynthesis essential for tumor proliferation (Khare et al., 2021; Sciacovelli et al., 2022; Table 3).

##### 3.3.3.2 ARG2 (arginase 2)

ARG2 (arginase 2) is a mitochondrial enzyme that catalyzes the hydrolysis of arginine to urea and ornithine. ARG2 is broadly downregulated in ccRCC, rendering tumor cells incapable of synthesizing arginine endogenously, and thus dependent on extracellular arginine—creating a potential metabolic vulnerability (Ochocki et al., 2018). From a clinical perspective, alterations in ARG2 expression offer clear biomarker potential, as confirmed by metabolomic and proteomic analyses showing ARG2 downregulation in nearly all ccRCC patients (Ochocki et al., 2018).

Interestingly, despite its general downregulation, ARG2 function displays dual regulatory behavior. On one hand, ARG2 inhibits tumor proliferation and survival by depleting the cofactor pyridoxal phosphate (PLP) and inducing polyamine toxicity (e.g., putrescine accumulation); re-expression significantly suppresses tumor growth *in vivo* and *in vitro* (Ochocki et al., 2018; Coffey and Simon, 2024). In CA9-knockdown models, ARG2 upregulation promotes polyamine accumulation, inhibits the Warburg effect, and suppresses tumor proliferation and migration, suggesting ARG2 mediates a CA9-dependent metabolic suppression pathway (Xu et al., 2020a). On the other hand, ARG2 may also act as an immunoregulatory factor, contributing to immune evasion. ARG2 overexpression in tumor cells, Tregs, and stromal cells depletes L-arginine from the tumor microenvironment, suppressing T cell effector functions and weakening antitumor immunity (Weis-Banke et al., 2022; Table 3).

### 3.4 Biomarkers in other metabolism

#### 3.4.1 CP (ceruloplasmin)

Ceruloplasmin (CP) is a multicopper ferroxidase that catalyzes the oxidation of Fe^2+^ to Fe^3+^, thereby facilitating iron export via stabilization of the ferroportin–transferrin axis and limiting Fenton chemistry–driven oxidative stress, directly linking CP to cellular redox and metal homeostasis (Musci et al., 2014). In ccRCC, integrated single-nucleus RNA/ATAC sequencing with bulk proteogenomics identified CP as a tumor-cell marker: CP transcripts and chromatin accessibility are selectively elevated in tumor cells relative to proximal tubule cells, high CP levels track with higher histologic grade, and CP immunofluorescence co-localizes with CA9 in tumor regions (Wu et al., 2023d). Spatial transcriptomics demonstrated that CP expression is enriched in hyalinized stromal areas, and shRNA-mediated CP knockdown in ccRCC cells downregulated matrisome/EMT and inflammatory programs and reduced COL4A1, OSMR and TGM2, implicating CP in tumor–stroma crosstalk that supports matrix remodeling and invasion (Wu et al., 2023d). Prior work independently corroborates CP’s prognostic relevance: bulk tumor datasets show that CP overexpression associates with oncogenic pathway activation and poorer overall survival in ccRCC (Zhang et al., 2021a). Mechanistically, ccRCC-relevant transcription factors regulate CP (e.g., HIF1A, PAX8, with KLF9 nominated by motif and perturbation data), providing an epigenetic/transcriptional route by which hypoxia and lineage programs couple CP to metabolic adaptation in tumor cells (Wu et al., 2023d). Collectively, CP’s tumor-cell-restricted upregulation, redox/iron-handling function, and consistent prognostic signal support its use as a metabolism-linked biomarker in RCC (Wu et al., 2023d; Musci et al., 2014; Table 4).

**TABLE 4:** Functions and clinical biomarker value of molecules involved in other metabolism reprogramming in RCC.

Molecule	Function	Biomarker role or clinical value	References
CP	Multicopper ferroxidase that oxidizes Fe^2+^ to Fe^3+^, regulating iron export and redox balance.	Tumor-cell-specific upregulation in ccRCC; correlates with high grade and poor prognosis; linked to EMT and matrisome remodeling; potential prognostic and metabolic biomarker.	Wu et al. (2023d), Zhang et al. (2021a)
PCSK6	Calcium-dependent serine endoprotease that activates pro-proteins including growth factors and matrix enzymes	Tumor-cell-enriched in ccRCC; associated with high-grade tumors; contributes to invasion and metabolic adaptation; potential biomarker for aggressive disease	Wu et al. (2023d)

#### 3.4.2 PCSK6 (proprotein convertase subtilisin/kexin type 6)

PCSK6 is a secreted calcium-dependent serine endoprotease that activates diverse pro-proteins (including growth factors and matrix enzymes) at paired basic residues, positioning it to influence extracellular signaling and matrix dynamics that interface with tumor metabolism (Bassi et al., 2015). Using the same single-nucleus multi-omics framework in ccRCC, PCSK6 was identified as a tumor-cell-specific marker with higher expression in high-grade tumors, although single-cell level survival associations were not significant in that cohort, indicating a role in tumor aggressiveness rather than a stand-alone prognostic determinant (Wu et al., 2023d). External population-scale resources likewise annotate PCSK6 as a prognostic marker in kidney cancers (KIRC/KIRP), reinforcing clinical relevance across datasets even as effect direction and magnitude may vary by cohort and platform. Beyond RCC, functional studies in other solid tumors show that PCSK6/PACE4 promotes proliferation, EMT/invasion, and activation of downstream pathways (e.g., MAPK/ERK), supporting biological plausibility that elevated PCSK6 marks—and may help drive—metabolically demanding, invasive phenotypes (Wang et al., 2018). More broadly, recent RCC translational work underscores the utility of single-nucleus profiling to resolve therapy-sensitive subpopulations and pathway activities in patient-derived models, highlighting how transcriptomic markers can integrate with functional readouts for risk stratification and therapeutic design (Wu et al., 2023c). These data support PCSK6 as a tumor-cell-enriched, microenvironment-modulating protease with potential value as a metabolism-adjacent biomarker in RCC (Table 4).

## 4 Therapeutic strategies

### 4.1 HIF-2α inhibitors

The PAS-B domain of HIF-2α contains a unique hydrophobic cavity that confers “druggability,” spurring the development of several small-molecule HIF-2α inhibitors (Scheuermann et al., 2013). The first molecule to validate the druggability of HIF-2α was PT2399, which specifically disrupts the dimerization of HIF-2α with HIF-1β, thereby suppressing downstream target gene expression. PT2399 exhibited potent antitumor activity in the majority of patient-derived ccRCC xenograft models, surpassing the efficacy of sunitinib and maintaining activity even in sunitinib-resistant tumors (Chen et al., 2016).

Derived from PT2399’s lead structure, the next-generation compound PT2385 demonstrated improved preclinical potential. In its first-in-human trial, PT2385 showed favorable tolerability and pharmacokinetic profiles, achieving complete response in 2%, partial response in 12%, and stable disease in 52% of heavily pretreated advanced ccRCC patients (Courtney et al., 2018). However, its extensive glucuronidation metabolism limited drug exposure and therapeutic durability in clinical applications.

To address this limitation, a structurally optimized third-generation inhibitor, PT2977 (also known as Belzutifan, marketed as WELIREG), was developed. Belzutifan exhibits significantly improved pharmacokinetic stability and systemic exposure. Phase I and II clinical trials confirmed its excellent safety profile and robust antitumor activity. Notably, in patients with VHL-associated ccRCC, Belzutifan achieved an objective response rate of 49%, with most adverse events being grade 1–2 (Xu et al., 2019a; Jonasch et al., 2021a; Choueiri et al., 2021).

Belzutifan was approved by the FDA in 2021 for the treatment of VHL syndrome–associated ccRCC and has also demonstrated efficacy in sporadic ccRCC, although resistance remains a challenge. Emerging evidence suggests that mutations in HIF-2α, alterations at the HIF-1β binding interface, and p53 status may contribute to the development of resistance to HIF-2 inhibitors (Stransky et al., 2022).

Furthermore, novel drug delivery strategies have been explored to enhance efficacy. For instance, co-loading PT2385 with manganese into a nanoparticle platform (PMMF) significantly improved therapeutic outcomes in VHL-deficient ccRCC and activated STING pathway–mediated innate immune responses, highlighting a promising approach for combining metabolic and immunotherapeutic targeting (Fang et al., 2025).

### 4.2 GLUT inhibitors

Targeting GLUT1 has emerged as a novel therapeutic strategy in RCC. The small molecule STF-31 has been shown to significantly suppress RCC cell growth and induce apoptosis in both *in vitro* and *in vivo* models, suggesting that GLUT1 is a promising therapeutic target (Chan et al., 2011). Further studies have revealed that dual inhibition of GLUT1 and polycomb repressive complex 1 (BMI1/PRC1) synergistically induces the expression of endoplasmic reticulum (ER) stress–related genes, leading to tumor cell death. These findings highlight the therapeutic potential of combining metabolic and epigenetic targeting (Zhang et al., 2020).

BAY-876, a selective GLUT1 inhibitor, enhances cytotoxicity when combined with sunitinib in 786-O RCC cells (Hamadamin and Maulood, 2024). In immunocompetent animal models, BAY-876 was also found to amplify the immune-mediated clearance of low-glycolytic tumors by sensitizing them to TNF-α–induced cytotoxicity (Wu et al., 2023b). Interestingly, while GLUT1 is predominantly expressed in tumor cells, CD8^+^ T cells rely more heavily on GLUT3. As such, GLUT1 inhibition has minimal impact on CD8^+^ T cell function, but it can alleviate metabolic competition and lactic acid accumulation within the tumor microenvironment, thereby enhancing immune cell infiltration and antitumor efficacy (Wu et al., 2023b).

In the context of cancer immunotherapy, recent research has demonstrated that CAR-T cells engineered with enhanced GLUT1 expression exhibit improved memory stem-like phenotypes, metabolic fitness, and recall responses. These metabolic engineering strategies significantly improved tumor control, suggesting that targeting cellular metabolism may augment the clinical efficacy of CAR-T cell therapies (Shi et al., 2024).

### 4.3 FASN inhibitors

The traditional fatty acid synthase (FASN) inhibitor C75 has been shown to significantly induce G2/M phase cell cycle arrest and apoptosis in various RCC cell lines, such as 769P and Caki-1. Mechanistically, C75 suppresses cell proliferation and invasion by downregulating HER2, EGFR expression, and STAT3 phosphorylation, while upregulating the expression of p53 and p21 (Horiguchi et al., 2008). Additionally, C75 has demonstrated synergistic effects when combined with PI3Kα inhibitors (e.g., CYH33), jointly suppressing breast cancer growth and remodeling the tumor immune microenvironment, highlighting the promising application of FASN inhibitors in combined immunometabolic therapies (Sun et al., 2021).

TVB-2640, a novel orally available small-molecule FASN inhibitor, has shown favorable safety and target specificity in phase I clinical trials. Its adverse effects were mostly limited to reversible dermatologic and ocular toxicities, with no severe gastrointestinal or hematologic toxicities observed. As a monotherapy, TVB-2640 achieved a disease control rate (DCR) of up to 42%, which increased to 70% when combined with taxanes (Falchook et al., 2021). In multiple tumor models, including non-small cell lung cancer, ovarian cancer, and breast cancer, TVB-2640 effectively inhibited *de novo* lipogenesis and synergistically enhanced the efficacy of various chemotherapeutic agents (Kelly et al., 2023b). These findings underscore the substantial therapeutic potential of TVB-2640 in the treatment of ccRCC. Currently, clinical trials evaluating the efficacy of TVB-2640 in ccRCC are underway.

### 4.4 SLC1A5 and GLS inhibitors

DRP-104 (also known as sirpiglenastat), a functional inhibitor targeting both SLC1A5 and GLS, has demonstrated potent antitumor activity across various cancer models. DRP-104 is a prodrug of 6-diazo-5-oxo-L-norleucine (DON), engineered for tumor-selective activation and gastrointestinal deactivation, thus significantly mitigating the toxicity traditionally associated with DON treatment (Rais et al., 2022). Given the widespread glutamine addiction and high expression of SLC1A5 in ccRCC, DRP-104 holds considerable clinical potential. Its mechanisms—including metabolic inhibition, immune activation, and remodeling of the tumor microenvironment—offer new therapeutic avenues for ccRCC, particularly in combination with immunotherapy.

In multiple cancer types, DRP-104 suppresses glutamine metabolism to induce metabolic crisis and tumor cell death while reshaping the tumor microenvironment by enhancing CD8^+^ T-cell activity, reversing T-cell exhaustion, and promoting M1 macrophage polarization, thereby amplifying antitumor immunity (Yokoyama et al., 2022; Pillai et al., 2024). Notably, in KEAP1-mutant lung cancer and pancreatic ductal adenocarcinoma (PDAC), DRP-104 alone or in combination with ERK inhibitors has provided significant survival benefits (Encarnación-Rosado et al., 2024; Pillai et al., 2024). In head and neck squamous cell carcinoma, DRP-104 has been shown to induce lipid peroxidation and ferroptosis sensitivity through metabolic stress, suggesting its therapeutic synergy with GPX4 inhibition (Allevato et al., 2024). Additionally, DRP-104 has been shown to reprogram immunosuppressive metabolic networks, enhancing infiltration of TILs, NK cells, and NKT cells (Yokoyama et al., 2022).

Although no RCC-specific data are currently available, the broad-spectrum efficacy of DRP-104 across malignancies lays a solid foundation for its translational application in RCC.

CB-839 (telaglenastat) is a selective oral GLS inhibitor that has been extensively investigated in clinical trials for RCC-targeted therapy (Emberley et al., 2021). By blocking the conversion of glutamine to glutamate, CB-839 impairs the TCA cycle, imposing metabolic stress and weakening tumor cell survival. Preclinical studies have shown that CB-839 reduces the production of glutamine-derived metabolites and exerts synergistic antiproliferative effects when combined with cabozantinib or everolimus (Emberley et al., 2021).

In a phase I dose-escalation study, CB-839 demonstrated favorable safety and pharmacokinetics, with a recommended phase II dose of 800 mg twice daily. In this trial, RCC patients achieved a disease control rate (DCR) of 50%, suggesting potential efficacy as monotherapy (Harding et al., 2021). Subsequent phase Ib studies confirmed the tolerability and promising DCR of CB-839 when combined with cabozantinib (TelaC) or everolimus (TelaE) in patients with heavily pretreated mRCC, reporting DCRs of 100% in the TelaC arm and 95.2% among ccRCC patients in the TelaE arm (Meric-Bernstam et al., 2022). In the multicenter randomized CANTATA trial, although CB-839 combined with cabozantinib did not significantly prolong PFS compared to cabozantinib alone, its safety profile remained favorable (Tannir et al., 2022). Another randomized controlled trial revealed that CB-839 combined with everolimus extended PFS compared to placebo plus everolimus, supporting its synergistic interaction with mTOR inhibitors (Lee et al., 2022).

Moreover, CB-839 has been tested in combination with immune checkpoint inhibitors. A phase I/II trial showed that CB-839 combined with nivolumab was generally well tolerated, achieving an objective response rate (ORR) of 24% in immunotherapy-naïve ccRCC patients, although the response was limited in patients previously treated with PD-1/PD-L1 inhibitors (Gouda et al., 2025). Collectively, although CB-839 monotherapy yields modest efficacy, combination strategies hold further clinical development potential.

### 4.5 IDO inhibitors

Epacadostat, a highly selective oral inhibitor of indoleamine 2,3-dioxygenase 1 (IDO1), has been widely investigated in clinical trials across various cancers. In clinical studies involving metastatic renal cell carcinoma (mRCC), Epacadostat combined with the PD-1 inhibitor pembrolizumab demonstrated comparable efficacy to standard treatments such as sunitinib or pazopanib, without showing a clear therapeutic advantage (Lara et al., 2024). Although Epacadostat was able to reduce circulating kynurenine levels in mRCC patients, the suppression was insufficient to restore them to normal levels, suggesting incomplete inhibition of the IDO pathway. Moreover, preclinical and translational evidence indicates that resistance to IDO1 inhibition may not solely arise from inadequate enzymatic blockade but also from compensatory immunosuppressive pathways (Tang et al., 2021). For instance, parallel activity of tryptophan-2,3-dioxygenase (TDO2) and sustained kynurenine–aryl hydrocarbon receptor (AhR) signaling can maintain immunosuppression even when IDO1 is targeted (Opitz et al., 2011; Wu et al., 2023a). In addition, increased regulatory T-cell and myeloid-derived suppressor cell activity driven by the Kyn–AhR axis, together with alternative metabolic checkpoints such as the adenosine (CD39/CD73–A2A) and arginase pathways, may collectively undermine IDO1 inhibitor efficacy (Campesato et al., 2020; Zahavi and Hodge, 2023; Martí i Líndez et al., 2019). Furthermore, non-catalytic IDO1 signaling via its ITIM–SHP axis can sustain a tolerogenic program independent of enzymatic activity, potentially limiting the impact of purely catalytic inhibitors such as epacadostat (Panfili et al., 2023; Albini et al., 2017).

In early-phase clinical trials for other solid tumors—including melanoma, non-small cell lung cancer (NSCLC), and urothelial carcinoma (UC)—Epacadostat combined with pembrolizumab exhibited some antitumor activity. For instance, in melanoma, an objective response rate (ORR) of 55% was achieved (Mitchell et al., 2018). However, in other malignancies such as sarcomas, the combination therapy yielded a low response rate, with only 3.3% of patients achieving partial response at 24 weeks, and no significant alterations in tryptophan metabolism were observed, indicating inadequate IDO1 inhibition (Kelly et al., 2023a). Similarly, in a phase III trial for urothelial carcinoma, the ORR for Epacadostat plus pembrolizumab was similar to pembrolizumab monotherapy, with no evident benefit from the combination (Necchi et al., 2024).

Navoximod, an investigational small-molecule IDO1 inhibitor, has also attracted increasing attention in the treatment of various advanced malignancies. In patients with RCC and other late-stage solid tumors, Navoximod combined with the PD-L1 inhibitor atezolizumab showed acceptable safety and tolerability. Pharmacodynamic assessments indicated that Navoximod reduced plasma kynurenine levels in a dose-dependent manner, suggesting effective inhibition of IDO1 activity. While some patients achieved objective responses or disease stabilization, no definitive therapeutic advantage of the combination over monotherapy has been demonstrated thus far (Jung et al., 2019). In another study, Navoximod monotherapy in patients with recurrent or advanced solid tumors also showed a favorable safety profile and was generally well tolerated, though no significant tumor shrinkage was observed and disease stabilization occurred in a notable proportion of patients (Nayak-Kapoor et al., 2018). Furthermore, studies in Japanese patients with advanced solid tumors reported good tolerability and linear pharmacokinetics for both Navoximod monotherapy and combination with atezolizumab. In the combination group, the disease stabilization rate reached 80%, suggesting potential clinical utility when paired with immune checkpoint inhibitors (Ebata et al., 2020).

Taken together, current clinical evidence suggests that although Epacadostat and Navoximod are mechanistically sound and well tolerated in RCC and various solid tumors, their overall antitumor efficacy remains suboptimal. This may be attributed to insufficient IDO pathway inhibition or the complex immunosuppressive tumor microenvironment. Specifically, redundancy within immunoregulatory circuits—including compensatory TDO2 activity, persistent Kyn–AhR signaling, and activation of adenosine and arginase nodes—likely attenuates clinical responses and argues for biomarker-guided, multi-target strategies (e.g., dual IDO1/TDO2 or IDO1/AhR blockade) (Kang et al., 2024; Opitz et al., 2011). Future clinical efforts should focus on higher-dose strategies for Epacadostat, refined patient selection guided by precise biomarkers, and rational combination therapies—such as pairing with more potent immune checkpoint inhibitors or metabolic modulators—to better define and enhance their therapeutic potential.

In addition, several small-molecule inhibitors of indoleamine-2,3-dioxygenase 1 (IDO1), including KHK2455, LY3381916, and MK-7162, are under investigation, although clinical trials for RCC have not yet been conducted. LY3381916 is a potent, highly selective oral IDO1 inhibitor. A clinical study demonstrated that LY3381916 significantly suppressed IDO1 activity and enhanced CD8^+^ T-cell infiltration when administered alone or in combination with PD-L1 blockade. However, in patients with advanced solid tumors, its combination therapy showed limited clinical efficacy and was associated with a high risk of hepatotoxicity, suggesting that dosage optimization is needed to improve both safety and therapeutic outcomes (Kotecki et al., 2021).

### 4.6 Arginine deprivation therapy

ADI-PEG20 (pegylated arginine deiminase) exerts its antitumor effects by depleting extracellular arginine, thereby suppressing cancer cell proliferation. It has demonstrated promising preclinical and clinical potential across various malignancies. In clinical studies involving malignant pleural mesothelioma (MPM), non-small cell lung cancer (NSCLC), metastatic uveal melanoma (UM), and acute myeloid leukemia (AML), the combination of ADI-PEG20 with chemotherapy has shown notable antitumor efficacy, with a high proportion of patients achieving disease stabilization and significant prolongation of both OS and PFS. Particularly, patients with non-epithelioid MPM exhibited more pronounced therapeutic benefits (Szlosarek et al., 2024; Szlosarek et al., 2021; Chan et al., 2022; Tsai et al., 2021; Tsai et al., 2017). However, treatment efficacy may be limited by the development of neutralizing antibodies against ADI-PEG20 and re-expression of argininosuccinate synthetase 1 (ASS1) in some patients.

Although no clinical trials of ADI-PEG20 have been conducted specifically in RCC, emerging evidence indicates frequent loss of ASS1 expression in RCC—especially in the clear cell and sarcomatoid subtypes—suggesting a potential vulnerability to arginine deprivation. In such ASS1-deficient tumors, ADI-PEG20 may exert antitumor effects through a synthetic lethality mechanism, providing a strong theoretical rationale for its application. Given the known sensitivity of RCC to both immunotherapy and metabolic interventions, combinatory approaches involving ADI-PEG20 and immune checkpoint inhibitors or mTOR pathway inhibitors warrant further investigation.

## 5 Discussion

Renal cell carcinoma is a prototypical solid tumor that relies heavily on metabolic reprogramming to sustain its malignant phenotype. At the core of this process lies the loss of the VHL gene, which leads to aberrant activation of HIF-2α. This drives a systematic remodeling of multiple metabolic pathways, including the maintenance of the Warburg effect, lipid accumulation, and amino acid addiction. Specifically, VHL inactivation results in sustained expression of HIF-2α, which upregulates key metabolic regulators such as GLUT1, HK2, SLC1A5, and FASN, thereby promoting glucose uptake, fatty acid synthesis, and glutamine metabolism. These alterations collectively endow ccRCC cells with enhanced proliferative capacity, resistance to apoptosis, and immune evasion. Meanwhile, aerobic metabolic pathways—such as the TCA cycle, OXPHOS, and fatty acid oxidation—are systematically downregulated, establishing a characteristic “synthesis–utilization imbalance” metabolic pattern.

This review categorizes a series of metabolism-related biomarkers in RCC into nine functional groups, each showing promising potential for precision medicine applications: 1 Prognostic biomarkers (e.g., GLUT1, FASN, GLS); 2 Clinical staging indicators (e.g., HK2, CD36, SLC1A5); 3 Histological grading markers (e.g., ENO2, FABP5, GPX1); 4 Therapeutic targets (e.g., PFKFB3, SCD1, GPX4); 5 Therapeutic response monitoring markers (e.g., GLUT1, DHA, MCT1); 6 Drug resistance predictors (e.g., PFKFB4, PGK1, SLC1A5); 7 Risk stratification markers (e.g., TKT, FABP5, TDO2); 8 Immune-associated biomarkers (e.g., PGAM1, IDO1, ARG2); and 9 Imaging targets (e.g., GLUT1, SCD1). Notably, several of these molecules exhibit multidimensional functionality. For example, glycolytic enzymes such as GLUT1 and SCD1 serve not only as prognostic markers but also as imaging targets. Lipid metabolism enzymes like FASN, SCD1, and FABP5 are associated with both histological grading and therapeutic intervention. Amino acid metabolism-related molecules—including SLC1A5, GLS, and GPX4—are implicated in reshaping the immune microenvironment and have emerged as targets for novel metabolic inhibitors such as DRP-104 and CB-839. Additionally, molecules like TDO2 and ASS1 hold value in predicting resistance to immune checkpoint inhibitors and assessing immune responsiveness, thereby serving as critical indicators in drug screening and risk stratification. These multifaceted attributes underscore the notion that metabolic molecules should no longer be viewed merely as pathway intermediates but rather as foundational elements for precision therapeutic decision-making.

Importantly, the advent of multi-omics technologies has enabled a shift in our understanding of the RCC metabolic network—from single-gene observations to integrated, cross-layered system analyses. Recent studies have successfully incorporated transcriptomic, metabolomic, proteomic, and spatial transcriptomic data to characterize metabolic subtypes, such as the dedifferentiated clear cell RCC (DCCD-ccRCC) with distinct metabolic features, as well as to provide comprehensive proteomic insights into rare renal tumors (Hu et al., 2024; Li et al., 2024a). These advances offer a theoretical framework for improving the diagnosis, targeted therapy, and imaging-based prediction of atypical RCC subtypes. In line with this, multi-omics integration of patient-derived xenograft models has demonstrated that combined targeting of receptor tyrosine kinases and mTORC1/2 signaling effectively suppresses ERK-driven metabolic reprogramming and tumor growth, supporting the translational utility of pathway-level interventions in overcoming therapeutic resistance in ccRCC (Wu et al., 2023c). Multi-omics studies in other solid tumors likewise emphasize that the translation of metabolic biomarkers into precision oncology requires a stepwise validation process, including discovery in public datasets, external multi-cohort validation, and incorporation into clinically interpretable tools such as risk scores or imaging surrogates (Li et al., 2023b; Li et al., 2024c).

Moreover, emerging evidence suggests that certain canonical “metabolic enzymes” possess non-classical functions in specific contexts. For instance, HK2 has been shown to mediate PDHA1 phosphorylation, and ARG2 contributes to polyamine toxicity regulation and immune evasion—highlighting the need to explore metabolic proteins beyond their expression profiles and toward deeper functional characterization (Hu et al., 2024).

Despite these advances, several challenges remain to be addressed in the field of metabolic biomarker discovery and application in RCC. First, while multi-omics integration has illuminated previously unrecognized metabolic subtypes, the translation of such classifications into actionable therapeutic strategies is still in its infancy. Second, the non-canonical functions of metabolic enzymes highlight the complexity of metabolic signaling networks, underscoring the need for mechanistic studies that move beyond correlative expression profiling. Finally, the heterogeneity of metabolic dependencies within and across RCC subtypes calls for the development of spatially resolved, single-cell–level analytical approaches, as well as dynamic monitoring technologies, to capture temporal and spatial metabolic plasticity. Addressing these gaps will not only refine our understanding of RCC biology but also accelerate the implementation of precision metabolic interventions in clinical practice.

## 6 Conclusion

Metabolic reprogramming in RCC not only reshapes the intracellular biochemical landscape but also profoundly impacts the tumor microenvironment, immune modulation, and therapeutic responsiveness. Future research should prioritize the multidimensional biological roles of metabolic molecules, the integration of multi-omics data, and the clinical translation of these findings—particularly in the development of integrated imaging–therapeutic–prognostic biomarker systems. Such efforts are essential to advance metabolism-targeted strategies from mechanistic insights to real-world clinical applications.
